# Investigation on thermochemical energy network for efficient waste heat recovery

**DOI:** 10.1038/s41598-026-39243-7

**Published:** 2026-02-12

**Authors:** Mrinal Bhowmik, Alessandro Giampieri, Zhiwei Ma, Anthony Paul Roskilly

**Affiliations:** 1 Department of Engineering, Durham University, Durham, DH1 3LE UK; 2 Department of Mechanical Engineering, National Institute of Technology Manipur, Imphal, 795004 India

**Keywords:** Thermochemical energy network, Gaussian heating, Steady and regenerative thermal oxidiser profile, AI-based simulator, Energy science and technology, Engineering, Mathematics and computing

## Abstract

The performance of a thermochemical fluid (TCF)-based energy network is investigated for waste heat recovery and sustainable thermal management. An experimental TCF energy network was developed and tested under three different waste heating profiles, i.e. Gaussian, steady, and regenerative thermal oxidiser (RTO), across a range of air and solution flow rates and regeneration temperatures. An artificial intelligence-based multi-layer perceptron simulator was also developed to map the TCF energy network performance. Results demonstrate that higher air flow rates significantly enhance total energy recovery across a wide range of solution flow rates, with potential energy recovery effectiveness reaching around 30%. Increasing the heating temperature significantly improves the moisture recovery performance of the TCF network, while simultaneously reducing the sensitivity of the network to variations in the liquid-to-gas flow rate (L/G) ratio. At higher regeneration temperatures, humidity ratio differences up to 4.3 g/kg_da_ are achieved and the performance differences between L/G ratios become less pronounced. Across all profiles, the water removal to heat supplied (W/H ratio) decreases as the L/G ratio increases, indicating a consistent decline in performance at higher desiccant flow rates. The Gaussian heating profile offers the highest W/H ratio at lower L/G ratios compared to steady and RTO heating profiles. Further, the simulator demonstrates strong predictive accuracy for the TCF-based energy network at lower L/G ratios and under Gaussian and steady heating profiles, with low overall prediction errors. These findings provide essential insights for operating the TCF energy network, emphasising the importance of optimising working fluid operating conditions and regeneration temperatures.

## Introduction

 The global economy has expanded rapidly over the past few decades, primarily driven by industrial growth. However, rising industrial energy demand has created significant challenges related to primary energy consumption and carbon dioxide (CO_2_) emissions^1,2^. A large portion of the energy used in industrial processes is lost as waste heat. This heat is often released into the environment without recovery, leading to substantial energy waste^3^. Recovering waste heat can provide additional power, heating, or cooling without additional energy input. It can therefore improve energy efficiency and support emission reduction, playing a crucial role in decarbonisation^4^.

In recent years, many countries have increased their focus on industrial waste heat recovery. The U.S. Department of Energy evaluated several energy-intensive industries, including glass, cement, iron/steel, aluminium, metal casting, and ethylene production, that collectively consume about 8.86 EJ of energy annually, about 9% of the total U.S. energy consumption (∼92 EJ). Of this, 1.56 EJ of waste heat is released each year without recovery. Low-temperature waste heat below 230 °C represents about 60% of this total^5^. In China, waste heat accounts for 15–40% of the total energy input, with significant potential in industries like cement (41 GW_Th_), iron/steel (2.9 GW_Th_), and glass (1.8 GW_Th_). Waste heat below 150 °C comprises nearly half of the total recoverable waste heat^6^. Similarly, in the European Union, the total industrial waste heat potential is estimated to be 300 TWh/year, with about a third below 200 °C. Globally, low-temperature waste heat offers vast potential for recovery and reuse^7^.

High-temperature waste heat is relatively easy to recover because its higher energy levels can meet a wide range of user demands and can be used in power generation technologies such as steam turbines and organic Rankine cycles^8^. In contrast, low-temperature waste heat presents more challenges due to its lower energy levels, limited user demand, and inefficient heat-to-power conversion. Furthermore, waste heat is often produced in various profiles, for example, Gaussian, steady, regenerative thermal oxidiser (RTO), and stepwise heating, which can complicate recovery efforts. Because low-temperature waste heat is close to ambient temperature, identifying ways to effectively use this abundant energy source is crucial.

To address these challenges, researchers have explored various approaches, with thermochemical fluid (TCF) energy networks emerging as a promising solution. The earliest uses of TCFs for energy storage can be traced back to Kessling et al.^9,10^. In recent years, various strategies have been developed for thermochemical energy systems using TCFs. Geyer et al.^11^ estimated that the thermochemical networks approach could achieve significant energy savings, particularly in industrial drying applications (about 85%). In a later study, Geyer et al.^12^ further explored the technological and economic feasibility of thermochemical district networks, finding that humidity-related applications were the most promising, although space heating and cooling could also benefit. Quinnell et al.^13,14^ developed a single tank design capable of simultaneously storing water, diluted, and concentrated TCFs for both sensible heat and thermochemical energy storage. This system combines the ability to store thermal energy using desiccant solutions with the transport of thermochemical energy across various distances, although experimental data remains limited.

Burch et al.^15^ conducted an analytical study on a zero-energy community that incorporated a large solar central plant and liquid desiccant (LD) technology to provide space heating, hot water, and cooling for residential and commercial buildings. This system showed cost savings in distribution due to reduced piping sizes compared with conventional district heating/cooling networks. Delwati et al.^16^ developed a dynamic simulation model to evaluate the technical, economic, and environmental viability of a large-scale TCF network in Hasselt, Belgium. The results demonstrated the advantages of TCF networks over water-based district heating systems, particularly for long-distance heat transport. While TCF networks show substantial potential for waste heat valorisation, experimental data and control strategies remain limited, although some control strategies for conventional LD systems are available in the literature^17–19^. A comparison of representative desiccant-based thermochemical energy studies with the present work is presented in Table 1.

On the other hand, promising potential has also been identified in using offline system characterisation based on data-driven artificial intelligence (AI) platforms. This approach is valuable for developing agile, adaptive, and efficient multidimensional models to describe dehumidifier performance within a specific parametric design space. Researchers have successfully employed AI-based models to predict real-time performance parameters in various engineering fields. Rai et al.^20^ found that AI strategies provide greater accuracy than conventional parametric regression techniques, such as response surface modelling, particularly when dealing with datasets containing high levels of uncertainty. The ability of AI systems to handle highly non-linear behaviour is crucial for capturing typical dehumidifier performance patterns.

In the context of AI modelling, Gandhidasan and Mohandes^21^ constructed an artificial neural network (ANN) model to capture the correlation between inlet and outlet parameters of a packed adiabatic LD dehumidifier. The model used lithium chloride (LiCl) as the desiccant and had a dehumidifier height of 0.6 m with polypropylene packing, providing a specific area of 210 m²/m³. The inputs to the ANN architecture included air parameters (temperature, mass flow rate, and specific humidity), solution parameters (temperature, mass flow rate, and concentration), inlet cooling water temperature, and a dimensionless temperature ratio. The predicted outputs included desiccant solution concentration and temperature, and the rate of water condensation. Similarly, Shafei et al.^22^ used a multi-layer ANN architecture to predict the performance of a LD dehumidification system. Singh et al.^23^ employed Mamdani-type fuzzy logic models to estimate the moisture condensation rate during air dehumidification.

As shown in Table 1, the literature review showed that most studies on waste heat recovery have focused on systems with fixed heating profiles. To the best of the authors’ knowledge, a significant research gap remains in testing the performance of TCF energy networks under varying heating profiles, and no reliable simulator exists for predicting network performance. This gap presents an opportunity to optimise the efficiency of TCF systems across diverse industrial conditions.

This study includes both experimental and computational analyses. The experimental component involves controlled testing under steady, Gaussian, and RTO heating profiles to determine the network’s efficiency and resilience. The Gaussian profile offers insights into system behaviour under gradually varying temperatures, the steady profile evaluates performance under constant heating conditions, and the RTO profile assesses the impact of rapid thermal fluctuations. The real-time performance of the network with TCF is monitored throughout. The computational aspect involves developing and validating an AI-based simulator to predict the network’s dynamic performance under various thermal scenarios. Together, these approaches aim to provide comprehensive insights into the optimisation and operational strategies of TCF energy networks for waste heat utilisation, contributing to the advancement of sustainable energy recovery solutions.

The primary objectives of this study are to:

- Evaluate the dynamic performance of the network under different heating profiles, including steady, Gaussian, and RTO heating profiles.
- Develop and implement a systematic methodology for evaluating heat recovery performance by the TCF within the network.
- Construct a desiccant performance simulator using an AI-based multi-layer perceptron model to predict system behaviour under different heating profiles.

The current study is organised as follows. Section 2 details the experimental setup and procedures, addresses uncertainties, and describes the waste heating profiles used in the experiments, along with the modelling of the TCF-based energy networks. Section 3 focuses on evaluating the efficiency of the TCF-based energy networks, covering moisture efficiency, variations in humidity ratio, and the amounts of water removed or heat supplied, and providing a basis for assessing the network performance. Section 4 presents the outcomes of the study, detailing the performance of the TCF energy network under different heating profiles, including steady, Gaussian, and RTO heating. This section includes a comparative analysis of these profiles and discusses modelling results for dynamic performance. Section 5 summarises the study’s contributions, highlights limitations, and suggests directions for future work.

**Table 1:** Comparison of representative desiccant-based thermochemical energy studies with the present work.

Authors	Application/System Type	Heating Profile	Operating Conditions	Methodology	Performance Metrics	Key Limitation Addressed in Present Study
Gandhidasan & Mohandes [21]	Single packed-bed LD dehumidifier	Steady	Fixed inlet air and solution conditions	ANN-based component modelling	Outlet humidity ratio, condensation rate	Limited to steady-state, single-component analysis
Shafei et al. [22]	LD dehumidification system	Steady	Constant regeneration temperature	ANN-based prediction	Moisture removal rate, effectiveness	No consideration of dynamic or time-varying heat sources
Singh et al. [23]	LD air-conditioning system	Steady	Controlled laboratory conditions	Fuzzy logic modelling	Moisture condensation rate	Component-level focus without energy network integration
Quinnell et al. [13,14]	Thermochemical storage tank	Steady	Fixed charging/discharging conditions	Experimental and analytical	Energy storage capacity	No dynamic waste heat profile or regeneration control
Delwati et al. [16]	Thermochemical district network (simulation)	Steady	Assumed constant heat supply	Dynamic simulation	Energy efficiency, losses	Lack of experimental validation and transient profiles
Present study	TCF energy network	Steady, Gaussian, and RTO (dynamic)	Wide range of air/solution flow rates and temperatures	Experimental investigation + AI-based dynamic simulator	δωₐ(t), W/H, εₘ, dynamic performance prediction	Network-level experimental validation under realistic, time-varying industrial waste heat profiles

## Methodology

**Fig. 1:**
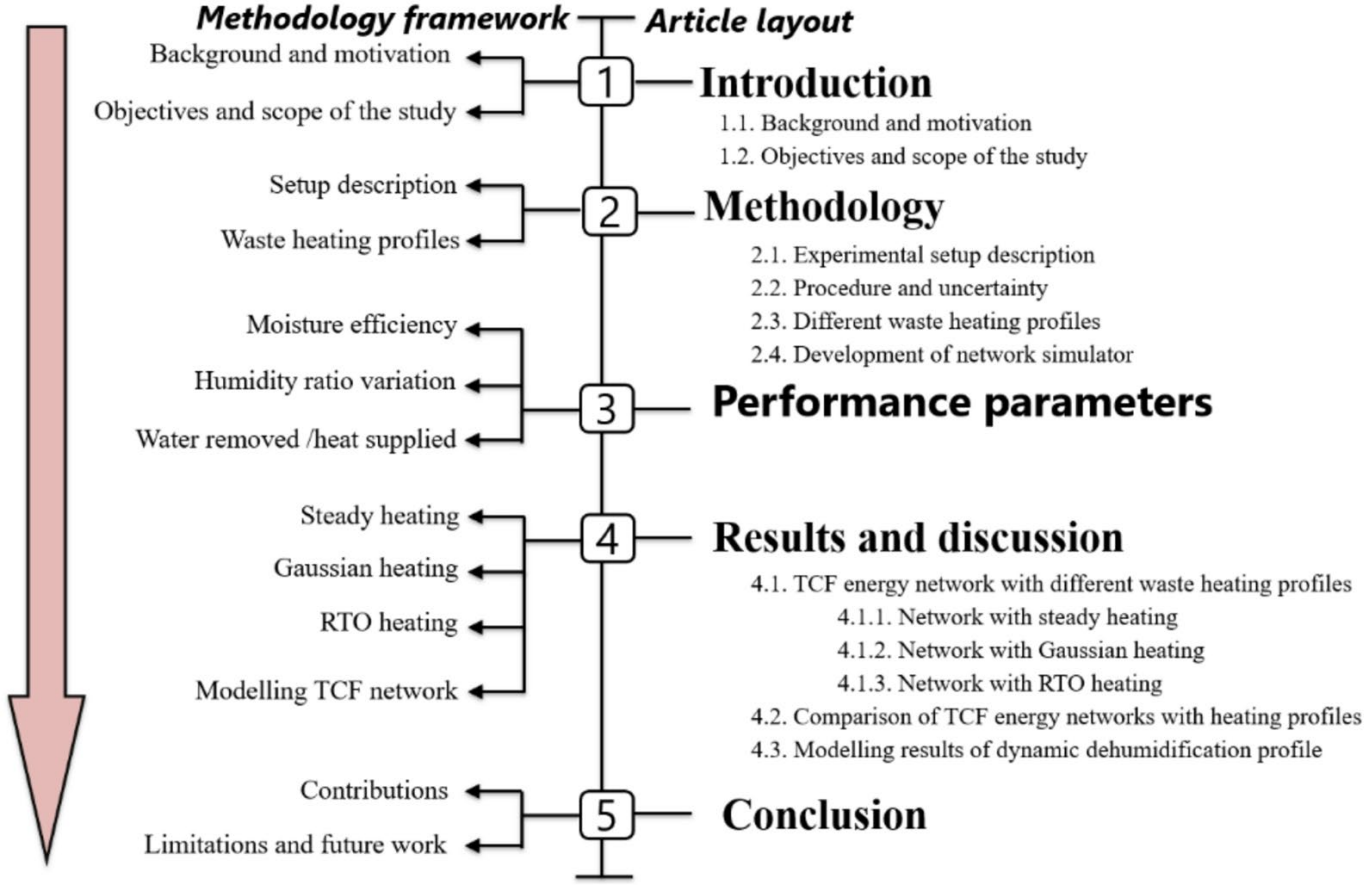
Workflow of the current research.

Comprehensive details of the methodology undertaken in the current study are presented in this section. It begins with a description of the experimental setup and the simulator development process. The different waste heating profiles are also defined, setting the stage for a results-driven analysis. The scope of the current research and its workflow is schematically illustrated in Fig. 1.

### Experimental set-up

**Fig. 2:**
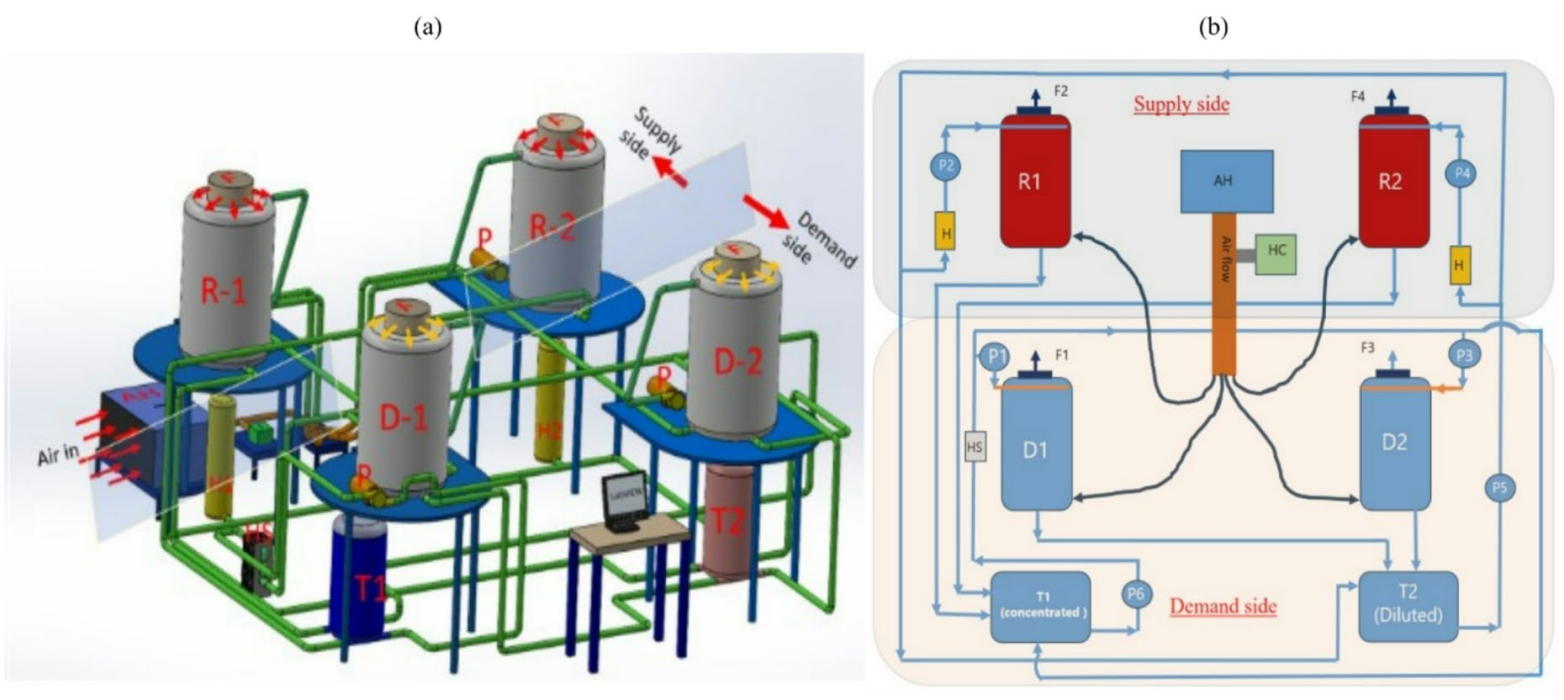
(**a**) 3D representation of developed TCF energy network and (**b**) its schematic layout (P: Pump, H: Heater, HS: Heat sink, D: Dehumidifier, R: Regenerator, F: Fan, T: Tank, AH: Air heater).

Fig. 2 shows a layout of the TCF energy network developed at Durham University, United Kingdom (54.7650° N, 1.5782° W). The experimental setup was designed to explore the potential of a TCF energy network to effectively recover heat from diverse waste heat sources for energy storage, as described in Giampieri et al.^24^.

**Fig. 3:**
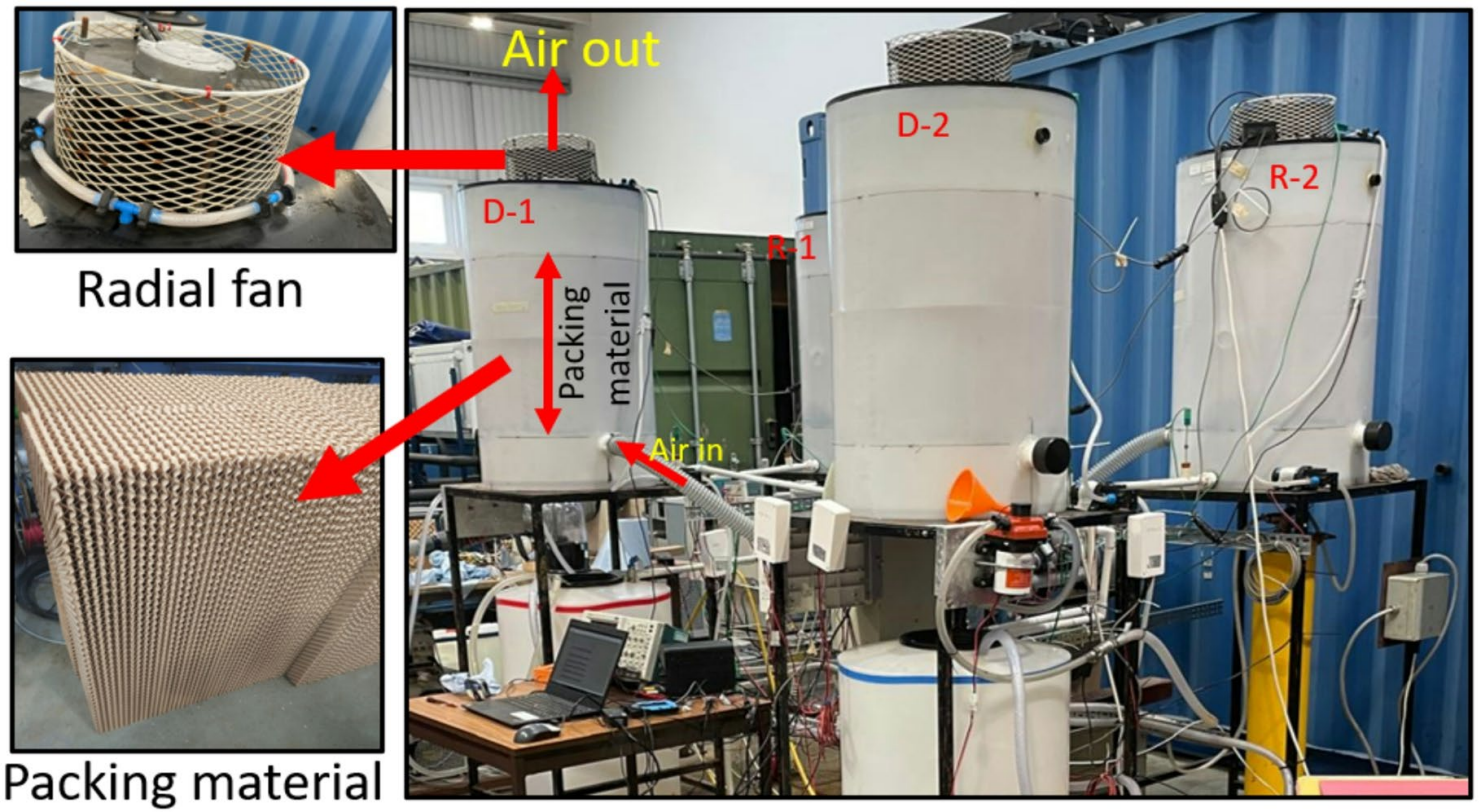
Pictorial view of the TCF energy network test rig (D: Dehumidifier, and R: Regenerator).

The network consists of interconnected components that facilitate the movement and treatment of air using a TCF, focusing on air dehumidification and desiccant regeneration. The system incorporates two dehumidifiers (D-1 and D-2) positioned on the demand side, and two regenerators (R-1 and R-2) on the supply side, all interconnected by a network of pipes and control elements. The setup includes two storage tanks (T1 and T2) that hold the TCF, ensuring a steady solution supply throughout the experiments. A heat sink (HS) is integrated to manage excess heat generated during the process, helping to maintain network temperature. Radial fans (F) are strategically placed to ensure a consistent airflow through the dehumidifiers and regenerators, optimising the contact between the air and the desiccant solution for efficient moisture transfer. The air heaters (AH) and humidity chamber (HC) are used to precondition the air by controlling its temperature and humidity before it enters the network, enabling precise control of initial conditions for experimental variations. To adjust humidity levels as needed, a water injector is utilised. If needed, a 10-litre boiling hot water tank supplies additional steam. The setup is equipped with a centralised control and monitoring system operated through National Instruments LabVIEW software. This system facilitates the assessment of waste thermal energy sources with varying temperature profiles and their application across a range of demand profiles. The interface allows precise adjustment of flow rates, temperatures, and TCF circulation in the network, ensuring reliable and accurate data collection throughout the experiments. The pictorial view of the TCF energy network experimental setup is shown in Fig. 3.

The technical specifications of the experimental test rig are listed in Table 2. The dehumidifier and regenerator chambers are identical in shape and size and contain cylindrical structured cellulose packing (PERICOOL 4545/7). A 6 mm thick thermal insulation layer of nitrile foam (Make: A-Flex) rubber was used to insulate the regenerator. This configuration minimised heat losses to the ambient environment under the investigated operating conditions. The dimensions of the packing chamber were determined based on the design guidelines outlined by Elsarrag et al.^25^.

**Table 2:** Technical specifications of the TCF network.

Parameter	Specifications
Packing material	Cellulose structured packing (PERICOOL 4545/7)
Packing diameter (Φ)	0.55 m
Packing height (β)	0.52 m
Flute height (δ)	0.007 m
Corrugation angle (θ)	45°
Flow pattern	Counter-flow
Fans	4
Solution pumps	6
Solution heaters	2 industrial immersion heaters
Solution heater capacity	18 kW
Storage tanks	2
Storage tank height	0.95 m
Storage tank diameter	0.55 m
TCF	Aqueous calcium chloride (CaCl_2_) (26–28 wt%)

### Experimental procedure and uncertainty

As shown in Fig. 2, air enters the network through the inlet marked as ‘Air in’ and is directed towards the dehumidifiers and regenerators. Once conditioned, the air is introduced into the TCF energy network. On the supply side, the TCF is exposed to heat provided by heaters H1 and H2. After heating, the TCF is sprayed over the packing material within the regeneration columns. The contact between the hot solution and the air releases moisture from the TCF, increasing its concentration. The network’s response to steady and variable heat sources is monitored using the National Instruments LabVIEW software. Temperature, humidity, and flow rates are collected to assess network performance. The thermal loads are also varied to simulate real-world scenarios, enabling a comprehensive analysis of network efficiency and effectiveness under different conditions.

The experimental setup and procedure inherently involve certain uncertainties, primarily due to variations in temperature, humidity, and flow measurements. The use of calibrated instruments aims to minimise these uncertainties, although environmental fluctuations and human error can still contribute. Temperature measurements are subject to uncertainty due to the precision limits of the thermocouples used, which have an accuracy of ± 0.5°C. Other instruments, including their accuracy and range, are listed in Table 3. The uncertainty associated with performance parameters is evaluated using the analytical approach proposed by Kline and McClintock^26^. For a performance variable ‘y’ estimated from a set of measured inputs (X₁, X₂, …, Xₙ), the combined uncertainty is denoted as Δy. Based on the instrument accuracies summarised in Table 3, the uncertainty of any performance-related parameter was computed using Eq. (1), following the methodology outlined by Kline and McClintock^27,28^.

1 $$\:\varDelta\:y=\sqrt{{\left(\frac{\partial\:{f}_{1}}{\partial\:{x}_{1}}\right)}^{2}{\left(\varDelta\:{x}_{1}\right)}^{2}+{\left(\frac{\partial\:{f}_{2}}{\partial\:{x}_{2}}\right)}^{2}{\left(\varDelta\:{x}_{2}\right)}^{2}+.......... +{\left(\frac{\partial\:{f}_{n}}{\partial\:{x}_{n}}\right)}^{2}{\left(\varDelta\:{x}_{n}\right)}^{2}}$$

2 $$\:\frac{\varDelta\:y}{y}=\sqrt{{\left(\frac{\partial\:{f}_{1}}{\partial\:{x}_{1}}\right)}^{2}{\left(\frac{\varDelta\:{x}_{1}}{y}\right)}^{2}+{\left(\frac{\partial\:{f}_{2}}{\partial\:{x}_{2}}\right)}^{2}{\left(\frac{\varDelta\:{x}_{2}}{y}\right)}^{2}+..........+{\left(\frac{\partial\:{f}{n}}{\partial\:{x}_{n}}\right)}^{2}{\left(\frac{\varDelta\:{x}_{n}}{y}\right)}^{2}}$$

Equation (1) defines *Δy* as the total propagated uncertainty, where *Δx₁* through *Δx*_n_ represent the uncertainties of each measured input. The uncertainty of the derived performance parameters was evaluated using the Kline and McClintock method. For a generic function $\:\mathrm{R}=f({\mathrm{y}}_{1},{\mathrm{y}}_{2},\dots\:,{\mathrm{y}}_{\mathrm{n}})$, the combined uncertainty is given by Eq. (2). Accordingly, the uncertainties of the derived performance parameters (as defined in Sect. 3) are expressed in Eqs. (3–5).

3 $$\:\delta\:(W/H)={\left[{\left(\frac{\partial\:(W/H)}{\partial\:\dot{m}a}\delta\:\dot{m}a\right)}^{2}+{\left(\frac{\partial\:(W/H)}{\partial\:\omega\:a,in}\delta\:\omega\:a,in\right)}^{2}+{\left(\frac{\partial\:(W/H)}{\partial\:{\omega\:}_{a,out}}\delta\:{\omega\:}_{a,out}\right)}^{2}+{\left(\frac{\partial\:(W/H)}{\partial\:Q}\delta\:Q\right)}^{2}\right]}^{1/2}$$

4 $$\:\delta\:{\epsilon\:}_{m}={\left[{\left(\frac{\partial\:{\epsilon\:}_{m}}{\partial\:{\omega\:}_{a,in}}\delta\:{\omega\:}_{a,in}\right)}^{2}+{\left(\frac{\partial\:{\epsilon\:}_{m}}{\partial\:{\omega\:}_{a,out}}\delta\:{\omega\:}_{a,out}\right)}^{2}+{\left(\frac{\partial\:{\epsilon\:}_{m}}{\partial\:{\omega\:}_{a,eq}}\delta\:{\omega\:}_{a,eq}\right)}^{2}\right]}^{1/2}$$

5 $$\:\delta\:\left(\delta\:{\omega\:}_{a}\right)={\left[{\left(\frac{\partial\:\left(\delta\:{\omega\:}_{a}\right)}{\partial\:{\omega\:}_{a,in}}\delta\:{\omega\:}_{a,in}\right)}^{2}+{\left(\frac{\partial\:\left(\delta\:{\omega\:}_{a}\right)}{\partial\:{\omega\:}_{a,out}}\delta\:{\omega\:}_{a,out}\right)}^{2}\right]}^{1/2}$$

Based on the accuracy of the measuring instruments, the uncertainty of the values calculated using Eqs. (3), (4) and (5) are ± 3.6%, ± 3.8% and ± 3.3% respectively.

**Table 3:** Specifications and accuracy details of measurement instruments.

Parameter	Device	Type	Accuracy	Range
Air dry-bulb temperature and humidity	Humidity and temperature transducer	HTM2500LF	± 3%	T = −40–85 °C RH = 0–100%
Air flow rate	Hotwire thermo-anemometer	Testo 405I	± 0.1 m/s	0–20 m/s
Solution temperature	Thermocouple	K-type	± 1.5 °C	0–350 °C
Density	Pycnometer with thermometer	BZP-FW-50	± 0.005 kg/m^3^	0–3 g/cm³ (T = 0–50 °C)

### Different waste heating profiles

**Fig. 4:**
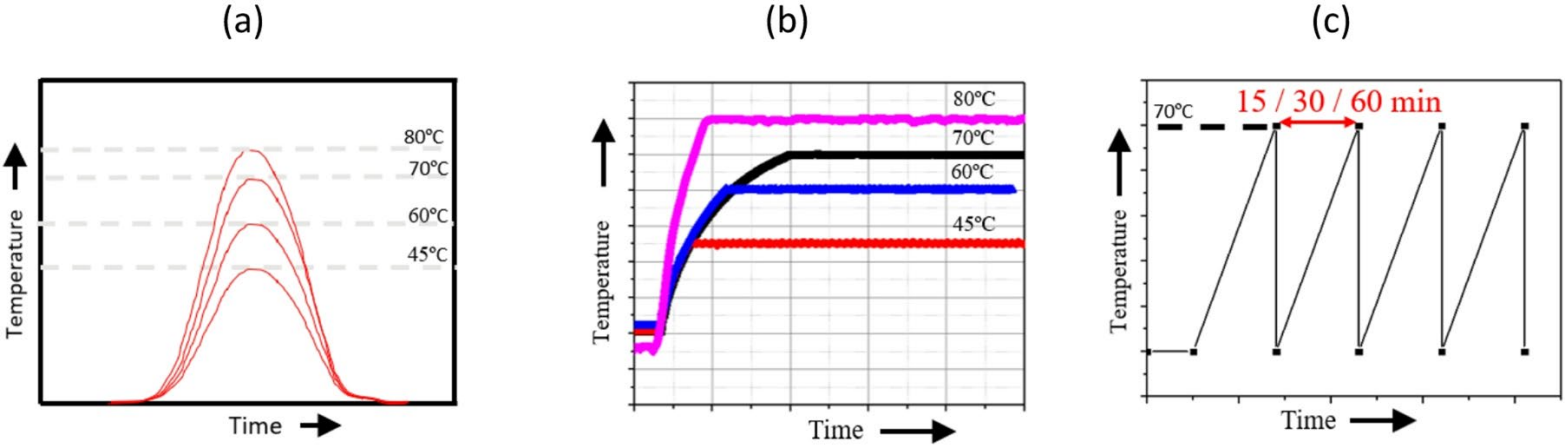
Different waste heating profiles: (a) Gaussian, (b) steady, and (c) RTO.

Three distinct heating profiles were investigated in this study, i.e. Gaussian, steady, and RTO, as depicted in Fig. 4. These profiles were emulated to assess the impact of different temperature-time characteristics on the performance and efficiency of the TCF energy network rig.

The Gaussian heating profile, as shown in Fig. 4 (a), is characterised by a bell-shaped temperature curve that rises and falls symmetrically around the peak. The time-varying Gaussian heating profile can be mathematically expressed in Eq. (6):

6 $$\:\mathrm{T}\left(\mathrm{t}\right)=\mathrm{A}\cdot\:\mathrm{e}\mathrm{x}\mathrm{p}\left[-\frac{{\left(\mathrm{t}-{\mathrm{t}}_{\mathrm{c}}\right)}^{2}}{2{{\upsigma\:}}^{2}}\right]$$

where *A* is the peak temperature (Gaussian) amplitude, *t*_*c*_ is the centre time corresponding to the maximum heat input, while *σ* represents the temporal spread of the profile with σ^2^ its variance. This profile simulates a scenario where the temperature gradually increases to a maximum value and then decreases symmetrically, representing a heating pattern that peaks and tapers off in a controlled manner. The experiment involves subjecting the system to a Gaussian temperature curve with peak values at 45 °C, 60 °C, 70 °C, and 80 °C. The rise and fall times are managed to replicate a smooth and consistent heating cycle, enabling analysis of the network’s response to varying peak temperatures and their gradual decline.

The steady heating profile, shown in Fig. 4 (b), maintains a constant temperature over a prolonged period. This profile involves heating the system to a set temperature and keeping it steady for the duration of the experiment. The steady temperatures tested are 45 °C, 60 °C, 70 °C, and 80 °C. This profile evaluates the network‘s performance under stable thermal conditions, enabling an assessment of equilibrium behaviour and the efficiency of the regeneration processes when exposed to consistent heating.

The RTO heating profile, shown in Fig. 4 (c), involves rapid and repetitive heating cycles to a peak temperature of 70 °C followed by cooling to a baseline temperature. Each cycle is characterised by heating the system quickly to 70 °C and then allowing it to cool down, repeating the process for durations of 15, 30, and 60 min. The RTO-type heating profile can be represented as a periodic square-wave function in Eq. (7):

7 $$\:\mathrm{T}\left(\mathrm{t}\right)=\mathrm{B}\cdot\:\mathrm{sgn}\left[\mathrm{s}\mathrm{i}\mathrm{n}\right(2{\uppi\:}\mathrm{t}/{\uptau\:}\left)\right]$$

where *B* is the temperature amplitude, and *τ* is the cycle period, corresponding to the characteristic switching behaviour of regenerative thermal oxidisers. This profile mimics real-world scenarios where the system may experience sudden bursts of heat followed by cooling phases. The RTO profile analyses the system’s resilience and response to rapid thermal changes, providing insights into its thermal stability and ability to recover from repeated heating and cooling cycles. The waste heating profiles depicted in Fig. 4 would be achieved by maintaining the heater 3/3 seconds on/off, simulating a dynamic thermal environment. The 15–60 min duration refers to the industrial RTO operating cycle, whereas the 3/3 seconds on/off switching corresponds to the laboratory-scale control implementation used to emulate the cyclic thermal input. Each heating profile is controlled and monitored using the National Instruments LabVIEW interface to ensure precise temperature regulation and data acquisition.

#### Test conditions

The current experimental work adopts the Taguchi method to systematically explore the effects of multiple factors, such as the solution mass flow rate $(\dot m_s)$, air mass flow rate $(\dot m_a)$, and heater temperature (*T*_*H*_), on the network output. As part of the Design of Experiments (DOE), the Taguchi method is highly effective for analysing and optimising processes by simultaneously evaluating the combined influence of several variables. Unlike traditional experimental designs, where only one factor is varied at a time, the Taguchi method uses an orthogonal array to structure the experiments, allowing multiple factors to be varied together in a controlled manner^29^. The operating factors and their levels for the TCF network are listed in Table 4.

**Table 4:** Operating factors and their levels for TCF network.

Factors	Levels			
	1	2	3	4
ṁ_s_ (kg/s)	0.02	0.03	0.04	0.05
ṁ_a_ (kg/s)	0.07	0.10	0.13	0.14
T_H_ (ºC)	45	60	70	80

The chosen levels are based on the operational limits of the experimental facility and the practical ranges encountered in industrial waste-heat-driven liquid-desiccant systems. In addition, preliminary trial experiments were conducted to ensure stable and repeatable operation without desiccant carryover or flooding. The factor levels employed in the Taguchi design were selected to represent realistic and practically relevant operating conditions of thermochemical fluid energy networks. The upper and lower bounds of ṁ_a_ and ṁ_s_ were constrained due to the solution carryover issue. The regeneration temperature levels were chosen to reflect the availability of low-grade industrial waste heat. Intermediate levels were defined to ensure sufficient resolution of parameter interactions within the feasible operating envelope, as confirmed through preliminary trial runs. This approach ensures that the Taguchi design captures both system performance sensitivity and practical operational constraints.

In general, an orthogonal array is typically represented in the form Lₓ(hᶻ), where *L* indicates the Latin square designation, *x* denotes the total number of experimental runs, *h* corresponds to the number of levels, and *z* specifies the number of factors. Among various types, two-level standard orthogonal arrays are widely utilised in experimental design. Further details and applications of orthogonal arrays are documented in the literature^30–32^. Table 5 presents the L_16_ (4^4^) orthogonal array used in this study.

**Table 5:** Taguchi L_16_(4^4^) orthogonal array for experiments.

Test condition	ṁ_s_ (kg/s)	ṁ_a_ (kg/s)	T_H_ (ºC)
1	0.02	0.07	45
2	0.02	0.10	60
3	0.02	0.13	70
4	0.02	0.14	80
5	0.03	0.07	60
6	0.03	0.10	45
7	0.03	0.13	80
8	0.03	0.14	70
9	0.04	0.07	70
10	0.04	0.10	80
11	0.04	0.13	45
12	0.04	0.14	60
13	0.05	0.07	80
14	0.05	0.10	70
15	0.05	0.13	60
16	0.05	0.14	45

By employing the Taguchi method, these experiments are designed to cover all possible combinations of the levels for each factor. This approach saves time and resources while enabling systematic evaluation of factor interactions and their influence on process outcomes. The experimental matrix includes 16 test conditions, each specifying a unique combination of ṁ_s_, ṁ_a_, and T_H_. The T and ω of the inlet air were maintained within the ranges of 20–25 °C and 6.4–7.4 g/kg_da_, respectively, while the T_H_ was varied between 45 °C and 80 °C depending on the heating profile. The solution mass flow rate was controlled between 0.02 and 0.05 kg/s, and the air mass flow rate between 0.07 and 0.14 kg/s, corresponding to liquid-to-gas (L/G) ratios ranging from 0.12 to 0.72. The desiccant solution concentration was maintained within 26–28 wt%, with inlet solution temperatures between 20.2 °C and 22.7 °C. Structured cellulose packing was assumed to be fully wetted during operation following an initial transient period, ensuring stable and repeatable heat and mass transfer conditions throughout the experiments.

### Development of a network simulator

**Fig. 5:**
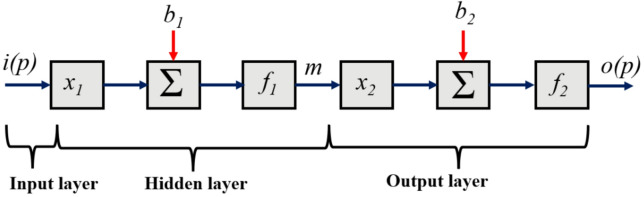
Simplified design of AI-MLP model.

An AI-based multi-layer perceptron (MLP) is considered for simulator development. A simplified model structure of the AI-MLP is shown in Fig. 5, consisting of input, hidden, and output layers. The simulator is developed using the MATLAB computational platform.

In an AI-based MLP, signals from the input layer propagate unidirectionally to the output layer through the network. The presence of at least one hidden layer of neurons facilitates mapping between input and output variables. The output response predicted by the model is formulated using Eqs. (8) and (9)^33^.

8 $$\:o\left(p\right)=\:{f}_{2}[{x}_{2}m\left(p\right)+\:{b}_{2}]$$

9 $$\:m\left(p\right)=\:{f}_{1}[{x}_{1}i\left(p\right)+\:{b}_{1}]$$

where *i(p)* and *o(p)* are the input and output vectors, respectively, while the state vector, *m(p)*, represents the output from the hidden layer. The linkage between the input and hidden layers is governed by the weight coefficient *x*_*1*_, while the connection between the hidden and output layers is defined by the weight coefficient *x₂*. The functions *f₁* and *f₂* denote the activation (transfer) functions applied within the hidden and output layers, respectively. The bias terms associated with the hidden and output layers are represented by *b₁* and *b₂*. Prior to training, the weights and biases are randomly initialised. During training, the TRAINLM algorithm iteratively updates these parameters using the gradient descent optimisation strategy^34–36^. A log sigmoid activation function is used in this study, as shown in Eq. (10).

10 $$\:\sigma\:\left(x\right)=1/[1\:+\:{e}^{\left\{-x\right\}}]$$

The number of hidden layers (1, 2, and 3) was tested during training. Although increasing the number of hidden layers enhances the network complexity, it does not always yield a notable gain in accuracy^37^. To ensure efficient training without compromising accuracy, a learning rate of 0.02 was selected. Key parameters influencing the TCF network’s performance were gathered, including the air mass flow rate (ṁₐ), humidity ratio (ωₐ), and temperature (Tₐ), and the mass flow rate of the solution (ṁₛ), its concentration (xₛ), and temperature (Tₛ). These parameters were then converted into nondimensional forms to simplify the modelling process: liquid-to-gas ratio (L/G) = ṁₛ/ṁₐ, humidity ratio (HR) = ωₛ/ωₐ, and temperature ratio (TR) = Tₛ/Tₐ.

#### Bayesian regularisation algorithm

Overfitting arises when the simulator becomes overly tailored to the training dataset, resulting in reduced predictive performance on unseen data. The complexity of the simulator is closely linked to the generalisation ability of the AI-MLP model; lower complexity typically enhances generalisation. As the simulator grows in size, the underlying mathematical representations become more intricate, thereby increasing complexity and reducing generalisation capability^38^. To address this challenge, the Bayesian regularisation algorithm is widely regarded as an effective approach for training AI-MLP models^39^. This method modifies the conventional sum of squared errors performance index by including a penalty term based on the sum of squared weights, which helps regulate model complexity (Eq. 6). By adding the sum-squared weight and therefore reducing the number of weight coefficients, the simulator can generate smoother interpolation across the training datasets, thereby mitigating the risk of overfitting. The corresponding regularisation term is represented in Eq. (11)^33^.

11 $$\:{\psi\:}_{re}=\:{\lambda\:}_{1}{\delta\:}_{D}+\:{\lambda\:}_{2}{\delta\:}_{w}$$

where *ψ*_*re*_ represents the objective function to be minimised. The term *δ*_*D*_ denotes the sum of squared differences between the actual and predicted outputs, while *δ*_*w*_ is the sum of squared network weights. The coefficients _*1*_ and _*2*_ serve as regularisation parameters within the objective function. By appropriately tuning the ratio of _1_ and _2_, the complexity of the simulator can be managed, thereby enhancing its ability to generalise system behaviour. In Bayesian-based simulators, the network weights are treated as random variables. The associated probability density function of the weights, along with the ratio of regularisation parameters, is inferred using Bayes’ theorem, as formulated in Eq. (12).

12 $$\:P\left(w|\varPhi\:,\:{\lambda\:}_{1},\:{\lambda\:}_{2},\:N\right)=\frac{P\left(\varPhi\:|w,\:{\lambda\:}_{1},\:N\right)P\left(w|{\lambda\:}_{2},\:N\right)}{P\left(\varPhi\:|{\lambda\:}_{1},\:{\lambda\:}_{2},\:N\right)}$$

where *w* denotes the vector comprising all weights and biases within the simulator, *Φ* represents the training dataset, and *N* defines the architecture of the simulator. A comprehensive explanation of the Bayesian regularisation technique used to enhance model generalisation is provided in Ref^38^. In addition, the overall complexity of the simulator is quantified by aggregating all weight and bias parameters utilised for data fitting^40^. In Eq. (13), the coefficients Ω₁ and Ω₂ act as weighting factors, assigning relative importance to the contributions of weights and biases in determining simulator complexity.

13 $$\:Ne{t}_{comp}={\varOmega\:}_{1}{\sum\:}_{1}^{p}\left({x}_{1,p}+{x}_{2,p}\right)+{\varOmega\:}_{2}{\sum\:}_{1}^{p}\left({b}_{1,p}+{b}_{2,p}\right)$$

As the dimensionality of the weight matrix exceeds that of the bias matrix, a weighting ratio of Ω₁:Ω₂ = 10:1 is adopted, emphasising the dominant role of weight coefficients in contributing to model complexity. During training, these coefficients are optimised to minimise the objective function presented in Eq. (11). Training is terminated when one of the following convergence criteria is met: a maximum of 1000 epochs (iterations), an error threshold of 0, a performance gradient of 10^− 7^ or a Mu (µ_a_) value of 10^10^. The root mean squared error (RMSE) and relative error (*e*_*rel*_) metrics are used to measure the external accuracy of the simulator’s prediction capability. The RMSE value, as shown in Eq. (14), reflects the quality of the data fit, while the relative error, as shown in Eq. (15), quantifies the prediction error for each individual data point.

14 $$\:R{\rm\:M}SE=\sqrt{\frac{1}{z}\left({\sum\:}_{i=1}^{z}{\left({\mu\:}_{e,i}-{\mu\:}_{p,i}\right)}^{2}\right)}$$

15 $$\:{e}_{rel}=\frac{{\sum\:}_{i=1}^{z}\left|{\mu\:}_{e,i}-{\mu\:}_{p,i}\right|}{{\sum\:}_{i=1}^{z}{\mu\:}_{e,i}}\cdot\:100$$

where z is the number of test cases, while $\:{\mu\:}_{p,i}$and $\:{\mu\:}_{e,i}$ are the predicted and experimental values, respectively.

## Performance parameters

The performance of the TCF energy network is evaluated using three key parameters: the change in air humidity ratio $\delta \omega_a$, the water removed per unit heat supplied (*W/H*), and the moisture effectiveness (*ε*_m_). These parameters provide insight into the efficiency and effectiveness of the system in terms of coupled heat and moisture transfer.

The parameter δω_a_ measures the difference between the outlet and inlet ω_a_. It indicates the amount of moisture desorbed to the air as it passes through the system, as shown in Eq. (16).

16 $$\:{\updelta\:}{\upomega\:}_a={{\upomega\:}}_{a,o}\left(t\right)-{{\upomega\:}}_{a,i}\left(t\right)$$

where *ω*_*a, o*_*(t)* and *ω*_*a, i*_*(t)* are the humidity ratios of the air over time at the outlet and inlet, respectively.

The parameter W/H represents the network’s efficiency in terms of water removal relative to the heat supplied. It is defined as the ratio of the total water vapour removed from the solution to the total heat supplied to the solution. The total heat supplied to the solution (*H*) can be calculated using Eq. (17).

17 $$\:\mathrm{H}={\int\:}_{0}^{{t}_{cycle}}\left[\dot{{m}_{s}}{C}_{p,s}{\Delta\:}{T}_{s}\left(t\right)\right]dt$$

where *t*_*cycle*_ is the experiment running time (assumed to be 40 min in this study), ṁ_s_ is the mass flow rate of the solution (kg/s), *C*_*p, s*_ is the specific heat capacity of the solution, and $\:{\Delta\:}{T}_{s}\left(t\right)$ is the temperature difference of the solution over time. Similarly, the total water removed from the solution can be estimated using Eq. (18). The procedure for estimating the total heat supplied to the TCF and the total water removed from the TCF is presented in Appendix A.

18 $$\:\mathrm{W}={\int\:}_{0}^{{t}_{cycle}}\left[\delta\:\omega\:_a\left(t\right)\right]dt$$

where δω_a_ (t) represents the rate of water vapour removal over time, while [ṁ_s_​ C_p, s​_ δT_s_​(t)] represents the rate of heat supply over time. Thus, the water removed per unit of heat supplied (W/H) can be calculated as shown in Eq. (19).

19 $$\:\frac{W}{H}=\frac{{\int\:}_{0}^{{t}_{cycle}}\left[\delta\:\omega\:_a\left(t\right)\right]dt}{{\int\:}_{0}^{{t}_{cycle}}\left[\dot{{m}_{s}}{C}_{p,s}{\Delta\:}{T}_{s}\left(t\right)\right]dt}$$

The quantities defined in Eqs. (17–19) were evaluated using numerical time integration of experimentally measured and time-resolved data. The integration was performed over the full experimental duration corresponding to each heating profile using a uniform time step consistent with the data acquisition rate. A trapezoidal integration scheme was employed to compute the time integrals of humidity ratio variation and heat input. Prior to integration, the raw data were filtered to remove sensor noise using a moving-average smoothing window, ensuring numerical stability without altering underlying trends. This approach provides accurate and repeatable evaluation of the derived performance parameters. All numerical integration and data smoothing were performed using OriginPro software.

All the thermodynamic functions related to the working fluids can be found in Appendix A. The integral expressions indicate the cumulative water removal and heat supply over the duration of the experiment. The parameter L/G is defined as the ratio between the solution and the air mass flow rate, as shown in Eq. (20).

20 $$\:\frac{L}{G}=\frac{\dot{{m}_{s}}}{\dot{{m}_{a}}}$$

The parameter εₘ is another key parameter for assessing the moisture transfer process. It is defined as the ratio between the change in ωₐ and the difference between the solution equilibrium humidity ratio, *ω*_*s*_, and the inlet ωₐ , as shown in Eq. (21):

21 $$\:{\epsilon\:}_{m}=\frac{{\updelta\:}{\upomega\:}_a\left(t\right)}{{{\upomega\:}}_{s}\left(t\right)-{{\upomega\:}}_{a,i}\left(t\right)}$$

where the equilibrium humidity ratio of the desiccant solution over time is derived from the equilibrium vapour pressure of aqueous solutions of CaCl_2_, which is obtained from Conde^41^.

## Results and discussion

In this section, the performance and behaviour of the TCF energy network are explored under three different waste heating profiles - steady, Gaussian, and RTO heating. A comparative assessment of these heating profiles is then presented to highlight their effects on the network. Finally, the modelling results for the dynamic dehumidification profile will be discussed, providing insights into its performance within the system.

### TCF energy network with different heating profiles

This section examines the behaviour of the TCF energy network under various waste heating profiles. The performance of the network depends on the type of heating applied, which affects efficiency and stability. Three specific scenarios are analysed: steady heating, Gaussian heating, and RTO heating. Each of these profiles has distinct characteristics, and their effects on the energy network are discussed in detail in this section.

#### Network with steady heating profile

**Fig. 6:**
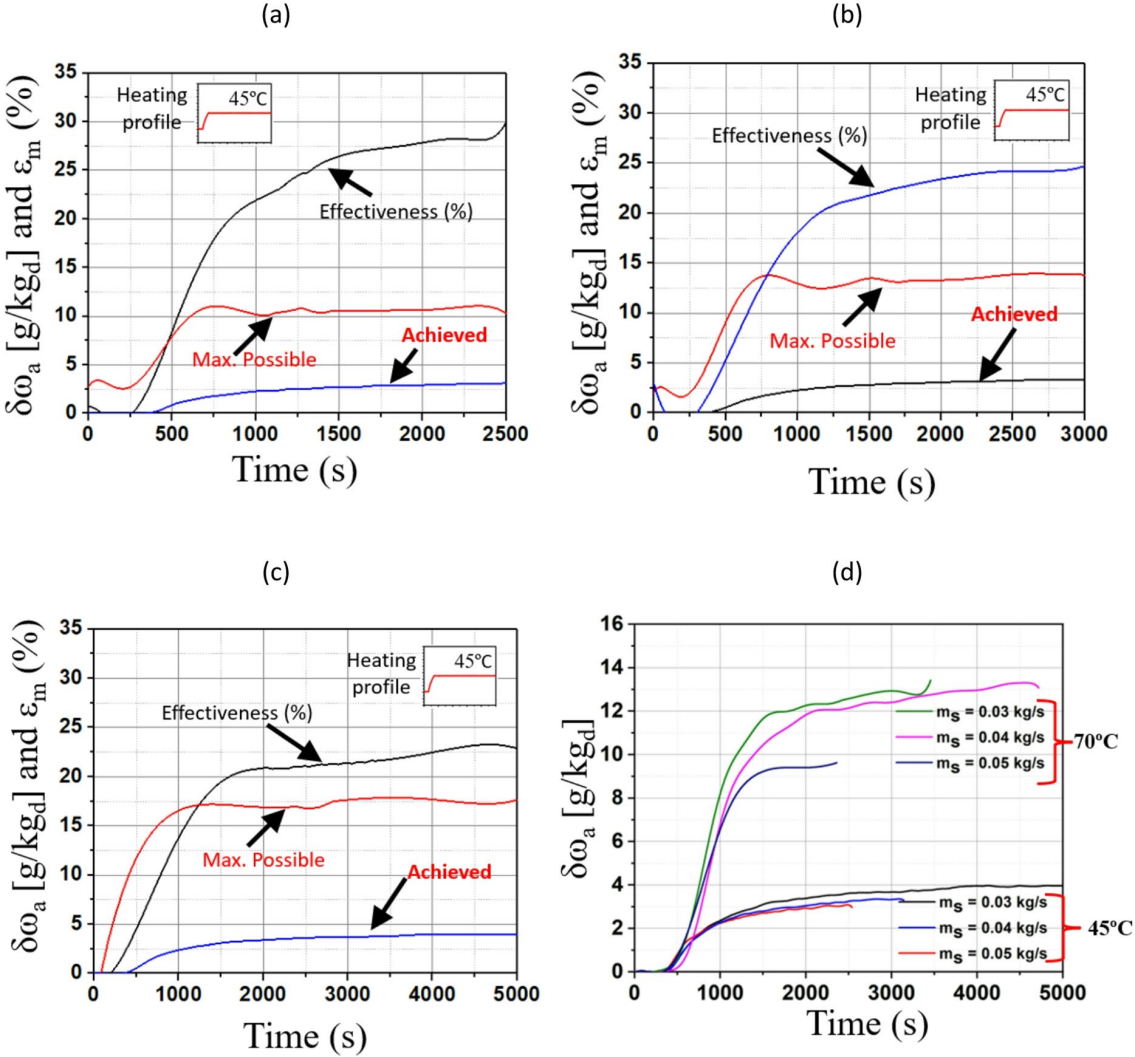
Dynamic performance of the TCF network with steady heating for ṁ_s_ of (**a**) 0.05 kg/s, (**b**) 0.04 kg/s, and (**c**) 0.03 kg/s, and for (**d**) T_H_ of 45 °C and 70 °C.

The dynamic performance details of the TCF energy network for waste heat recovery under different mass flow rates and heating profiles is shown in Fig. 6. Initially, the network exhibits a rapid increase in δω_a_ and εₘ due to the strong driving force between the desiccant and the air. At the start, as the desiccant begins to flow over the packing material, the surface is not fully wetted. Over time, the desiccant progressively wets the entire surface of the packing material, increasing the available area for mass transfer. As the air comes in contact with more wetted surfaces, greater moisture transfer occurs, leading to higher δω_a_ and εₘ.

Fig. 6 (a) shows the network’s performance with a steady heating profile of 45 °C and ṁ_s_ of 0.05 kg/s. The TCF energy network reaches a δω_a_ of around 3.2 g/kg_da_ after 2,500 s. The ratio of achieved and maximum possible humidity ratio variation represents the ε_m_ of the TCF energy network. This parameter steadily climbs to around 30%, while the maximum possible δω_a_ indicates that the network approaches a theoretical limit of around 10 g/kg_da_. However, a discrepancy remains between the achieved and maximum effectiveness due to system irreversibility and thermal losses in the network. Fig. 6 (b) presents the network’s response to a lower ṁ_s_ of 0.04 kg/s under the same heating profile, extended to 3,000 s. The εₘ steadily climbs to around 25%. The achieved δω_a_ is around 3.6 g/kg_da_, while the maximum possible δω_a_ increases (~ 14 g/kg_da_), indicating that the network has more potential to recover moisture at lower flow rates. Fig. 6 (c) illustrates the network behaviour with an even lower ṁ_s_ of 0.03 kg/s with a T_H_ of 45 °C, extending the observation period to 5,000 s. The εₘ steadily climbs to around 23.8%. Here, δω_a_ rises to nearly 4 g/kg_da_, while the maximum achievable ε_m_ increases (~ 17.5 g/kg_da_), indicating that the network has more potential to recover moisture at lower flow rates. This highlights that smaller flow rates can provide greater energy and moisture recovery potential. Fig. 6 (d) compares ṁ_s_ of 0.03, 0.04, and 0.05 kg/s under two heating profiles of 45 °C and 70 °C. The higher temperature significantly boosts δω_a_, especially for lower flow rates. At 70 °C, δω_a_ reaches around 13.3 g/kg_da_ for ṁ_s_ of 0.03 kg/s, whereas at 45 °C, δω_a_ reaches around 4 g/kg_da_ for the same ṁ_s_. The mass flow rate of 0.03 kg/s shows the larger moisture recovery, demonstrating that lower flow rates benefit more at higher temperatures.

**Fig. 7:**
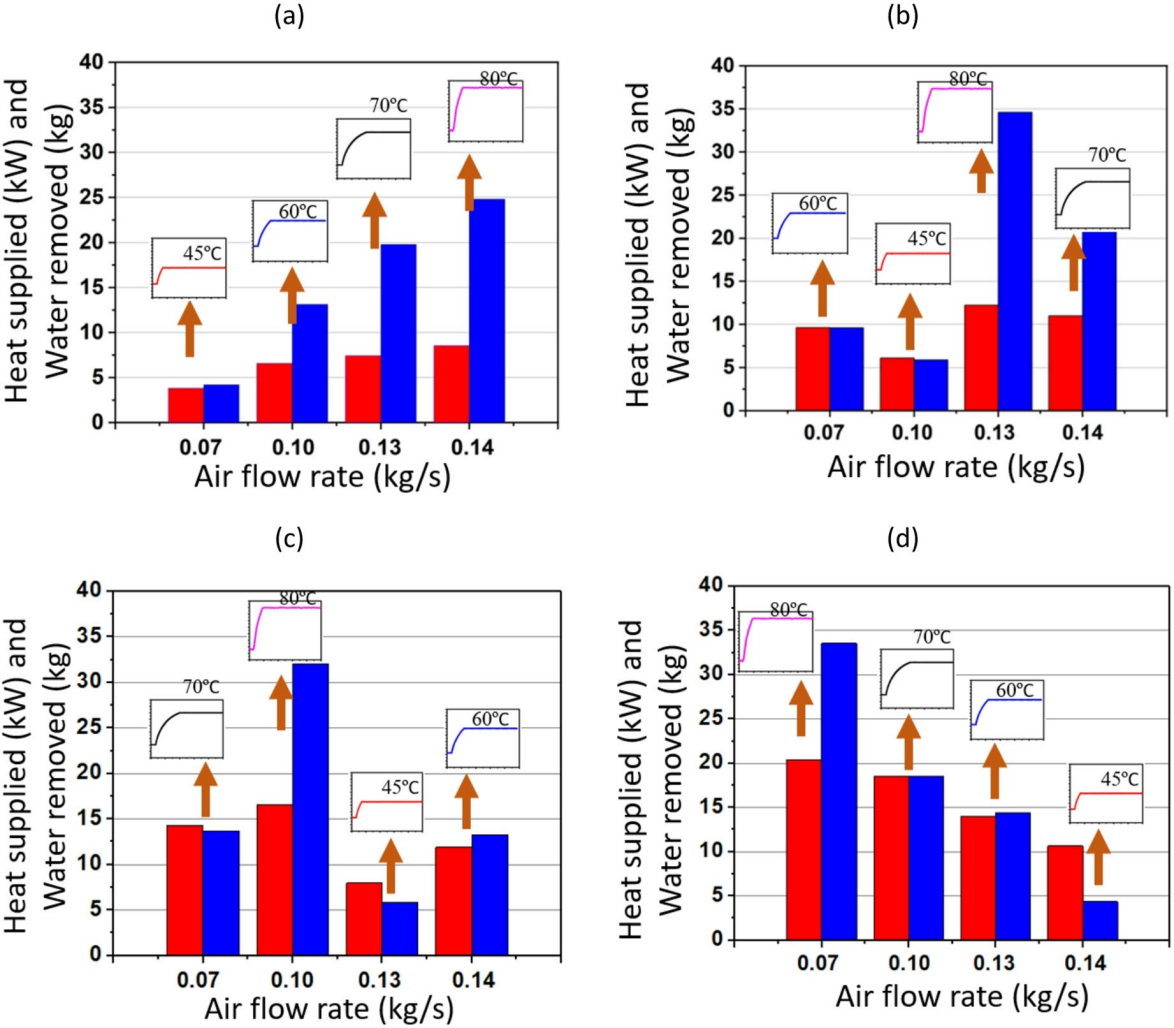
Performance of TCF energy network for ṁ_s_ of (**a**) 0.02 kg/s, (**b**) 0.03 kg/s, (**c**) 0.04 kg/s, and (**d**) 0.05 kg/s (where the heat supplied and water removed are represented by red and blue bars, respectively).

**Fig. 8:**
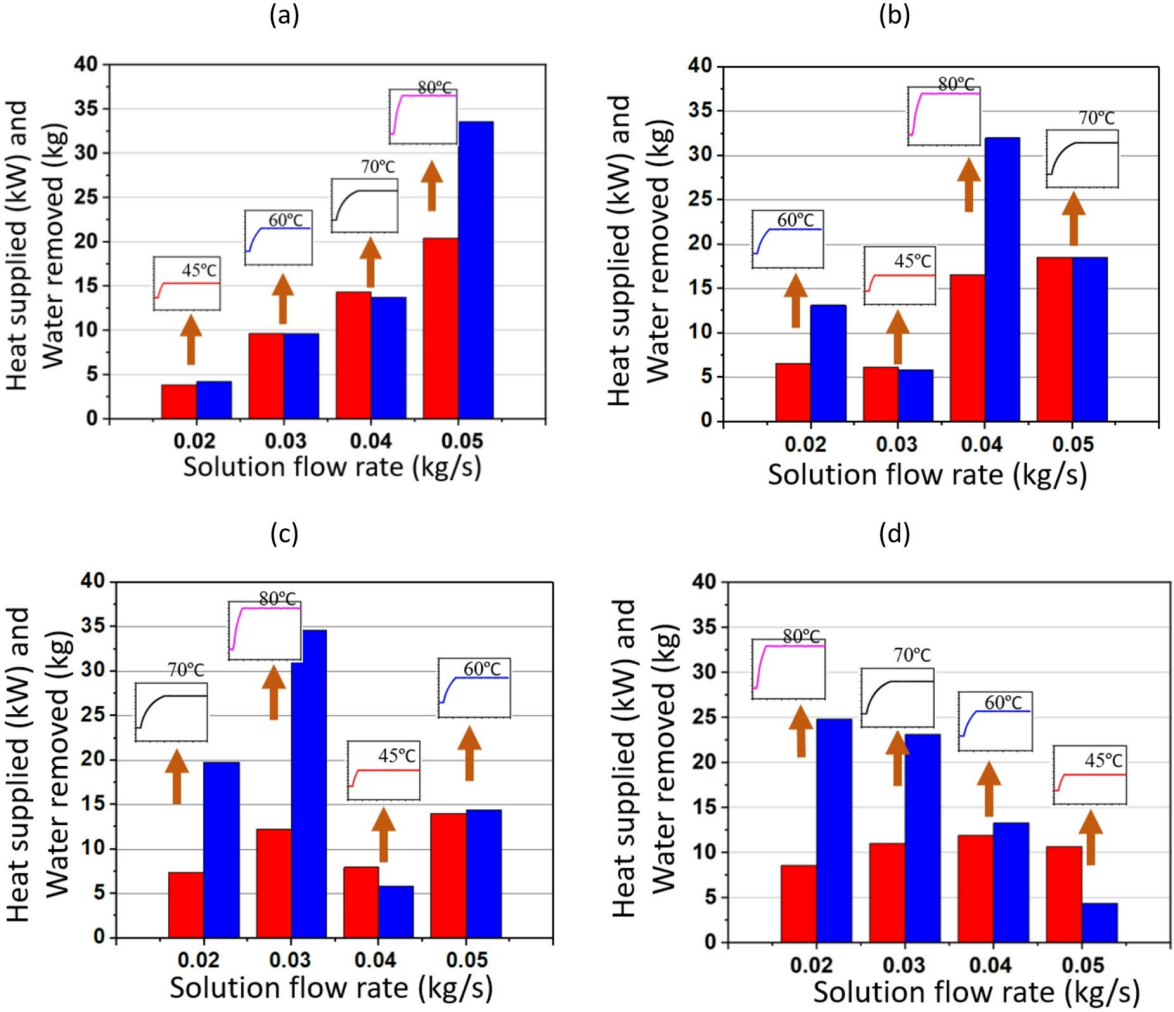
Performance of the TCF energy network for ṁ_a_ of (**a**) 0.07 kg/s, (**b**) 0.1 kg/s, (**c**) 0.13 kg/s, and (**d**) 0.14 kg/s (where the heat supplied and water removed are represented by red and blue bars, respectively).

Figs. 7 and 8 illustrate waste heat recovery under varying air and solution mass flow rates, focusing on changes in heat supplied and the corresponding water removal under different operating conditions. The fundamental mechanism involves the absorption of waste heat by the TCF. TCF absorbs moisture and is then released in the presence of waste heat with different magnitudes. In this network, the balance between air flow rate and solution mass flow rate is critical, as both govern the ability to recover and utilise energy efficiently. Higher flow rates generally promote greater heat transfer and water removal, although this relationship is modulated by the waste heat input temperature, which varies between 45 °C and 80 °C.

Fig. 7 focuses on the influence of different air flow rates. At ṁ_s_ of 0.02 kg/s, increasing the ṁ_a_ from 0.07 to 0.14 kg/s leads to higher heat supplied and greater water removal. As ṁ_a_ increases, more heat is transferred to the solution, raising the total heat supplied, and, in turn, the total water removed. This suggests that higher temperatures enhance the efficiency of moisture removal. For instance, at ṁ_a_ of 0.07 kg/s, the water removed at 0.02 kg/s solution flow rate is minimal, whereas it significantly increases at higher temperatures, particularly when the waste heat temperature is at 80 °C. This pattern is consistent across all the plots, indicating that higher temperatures enable greater water removal due to stronger convective effects.

On the other hand, Fig. 8 investigates the effect of ṁ_s_ while keeping ṁ_a_ constant. As ṁ_s_ increases from 0.02 to 0.05 kg/s, the amount of water removed increases with the heat supplied, although the trends depend on temperature. For ṁ_a_ of 0.07 kg/s, the amount of water removed amount increases significantly when ṁ_s_ is raised from 0.02 to 0.05 kg/s, particularly at higher temperatures (70 °C and 80 °C). A similar trend is observed for a given ṁ_s_, irrespective of ṁ_a_: higher waste heat temperatures correspond to greater water removal. Comparing performance at different temperatures, water removal is substantially higher at elevated temperatures (70 °C and 80 °C), as shown by the sharp increases in the blue bars in both Figs. 7 and 8. At lower temperatures (45 °C), system performance is noticeably poorer in terms of water removed because the driving potential for solution regeneration is lower, limiting the effectiveness of the TCF network.

**Fig. 9:**
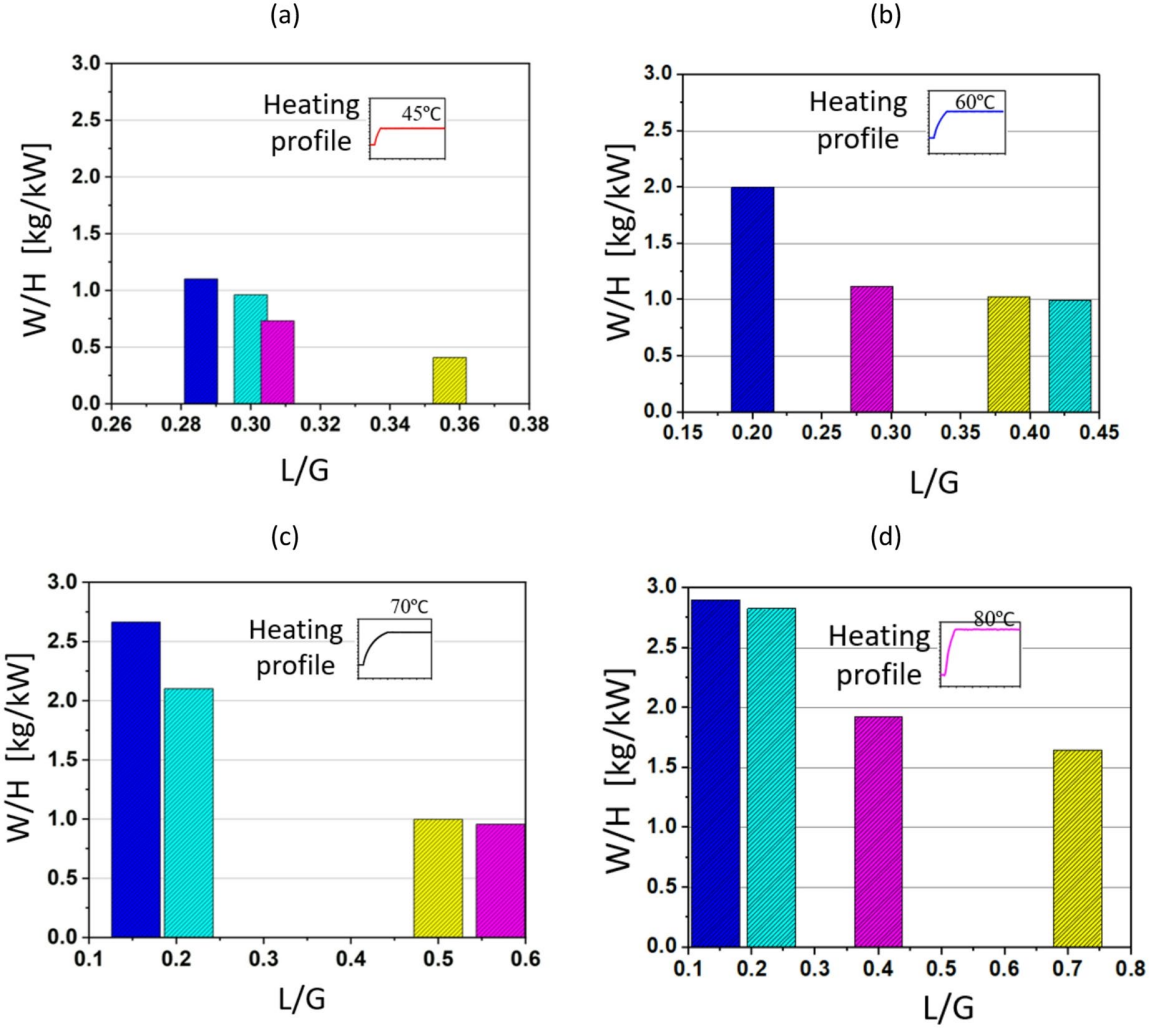
Performance of TCF energy network over L/G ratios with maximum steady T_H_ of (**a**) 45 °C, (**b**) 60 °C, (**c**) 70 °C, and (**d**) 80 °C.

The TCF energy network for waste heat recovery aims to optimise water removal from the desiccant solution using low-grade waste heat, as depicted in Fig. 9. The process relies on the thermodynamic interaction between air temperature, humidity ratio, and desiccant solution. By heating the solution at different temperature levels, the TCF network enhances moisture desorption from the desiccant, thereby improving the overall energy performance of the network. Lower L/G ratios generally correspond to higher water removal efficiencies per unit of heat input. This is because a lower L/G implies a lower value of ṁ_s_ for a given ṁ_a_. The balance between L/G ratios and temperature profiles therefore strongly influences the network’s overall capacity to recover waste heat.

Fig. 9 (a) presents the TCF network’s performance at a heating temperature of 45 °C. When the L/G ratio increases from 0.282 to 0.359, the W/H decreases significantly. At the lowest L/G ratio (e.g., 0.282), the W/H is 1.15 kg/kW, demonstrating the most efficient water removal. As the L/G ratio increases to 0.30 and 0.314, W/H drops to around 0.95 kg/kW and 0.75 kg/kW, respectively. This decline continues as the L/G ratio reaches 0.359, where W/H falls below 0.45 kg/kW. The results indicate that at a relatively low temperature of 45 °C, lower L/G ratios provide substantially higher water removal performance. As shown in Fig. 9 (b), the TCF network demonstrates improved performance across a wider range of L/G ratios when the waste heat temperature increases from 45 °C to 60 °C. Fig. 9 (b) shows a similar trend of decreasing W/H values as the L/G ratio increases from 0.20 to 0.43. At L/G of 0.20, W/H peaks at 2 kg/kW, although the increase is less pronounced than at 70 °C, as shown in Fig. 9 (c). As the L/G ratio increases to 0.42, the W/H value stabilises at around 1.0 kg/kW. In Fig. 9 (c), at a heating temperature of 70 °C, the network exhibits a broader range of performance, with a peak W/H of 2.6 kg/kW at an L/G ratio of 0.16. As the L/G ratio increases beyond 0.21, W/H decreases more sharply, falling to 1.0 kg/kW at a L/G ratio of 0.5 and further to around 0.9 kg/kW at a L/G ratio of 0.55. Fig. 9 (d) shows the network’s performance at 80 °C, where W/H remains high at around 2.75 kg/kW for a L/G ratio of 0.15. As in Fig. 9 (a–c), W/H decreases with increasing L/G ratio, reaching about 1.8 kg/kW at a L/G ratio of 0.4 and around 1.56 kg/kW at a L/G ratio of 0.72. These results suggest that the network can maintain reasonable performance even at higher L/G ratios when the heating temperature is increased, highlighting the benefit of utilising higher temperatures for improved moisture removal and heat recovery. At 80 °C, the TCF network demonstrates its best performance, indicating that the optimal operating point for maximising water removal lies in maintaining low L/G ratios while using elevated temperatures.

#### Network with Gaussian heating profile

**Fig. 10:**
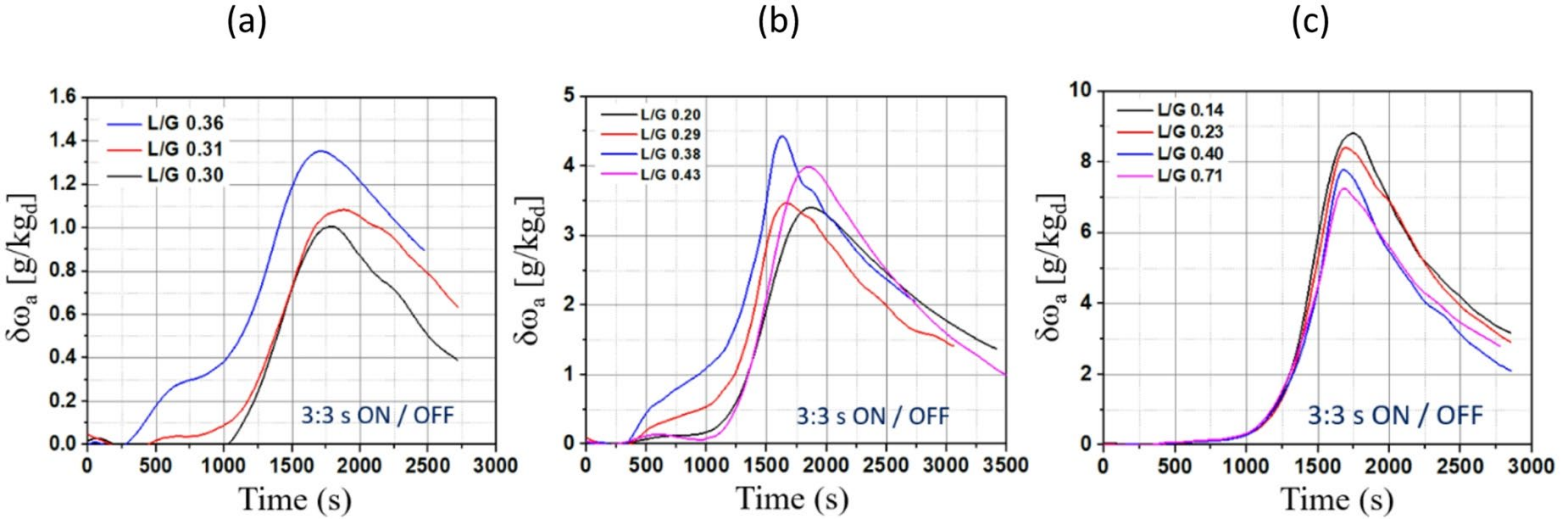
TCF Network performance with Gaussian-shaped waste heating profile with maximum T_H_ of (**a**) 45 °C, (**b**) 60 °C, and (**c**) 80 °C.

Fig. 10 illustrates the TCF network’s heat recovery performance under a Gaussian-shaped waste heating profile with varying L/G ratios and different T_H_. The TCF network uses intermittent heating cycles to maintain the desired waste heat profile. The δω_a_ is strongly influenced by both the L/G ratio and the maximum T_H_. It is also observed that the peaks occur within 1,500 to 2,000 s for all cases.

In Fig. 10 (a), δω_a_ peaks at around 1,800 s and then declines gradually when the maximum T_H_ is 45 °C. The L/G ratio significantly affects δω_a,_ with a L/G ratio of 0.36 yielding the largest increase (up to 1.38 g/kg_da_). In contrast, lower L/G ratios (0.31 and 0.30) result in more moderate peaks of approximately 1.1 g/kg_da_ and 1 g/kg_da_, respectively. This suggests that higher L/G ratios achieve greater δω_a_, as reflected in the higher peak values. Fig. 10 (b) shows the performance of the TCF network at a higher maximum temperature of 60 °C. The peak δω_a_ values are higher than in Fig. 10 (a), ranging from 3.2 g/kg_da_ to around 4.3 g/kg_da_. In Fig. 10 (c), the maximum T_H_ is set to 80 °C, and the range of L/G ratios extends from 0.14 to 0.71. Notably, the L/G ratio of 0.71 shows a lower peak, suggesting diminishing returns in δω_a_ as the air approaches saturation. Overall, the differences between the L/G ratios are less pronounced at this higher temperature. This indicates that increasing T_H_ both increases δω_a_ and reduces the sensitivity of the system to variations in the L/G ratio. This behaviour can be attributed to the higher energy input, which enhances the moisture release from the desiccant during regeneration, making the process more efficient overall, even at higher L/G ratios.

**Fig. 11:**
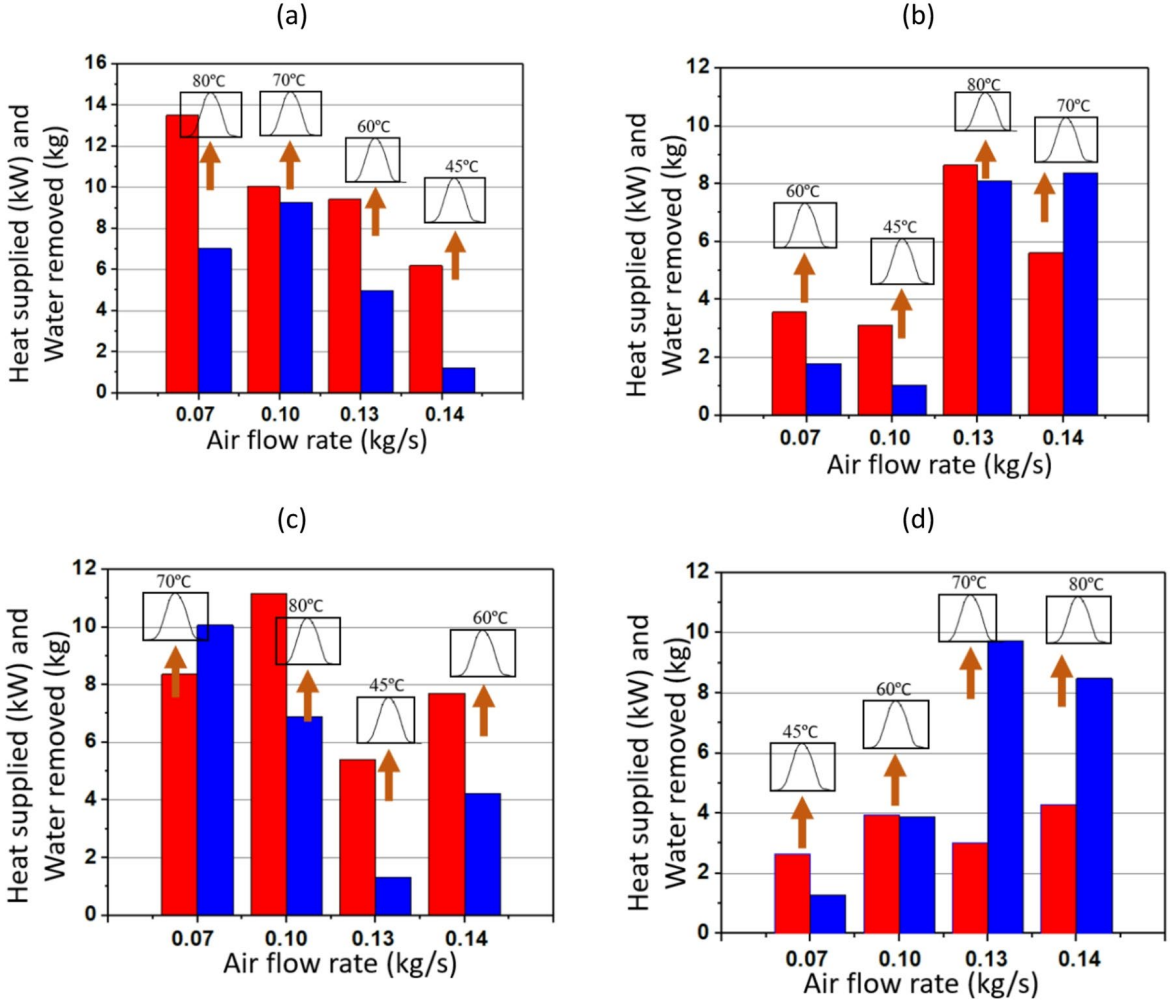
Performance of the TCF energy network with Gaussian heating profile for ṁ_s_ of (**a**) 0.05 kg/s, (**b**) 0.03 kg/s, (**c**) 0.04 kg/s, and (**d**) 0.02 kg/s (where the heat supplied and water removed are represented by red and blue bars, respectively).

**Fig. 12:**
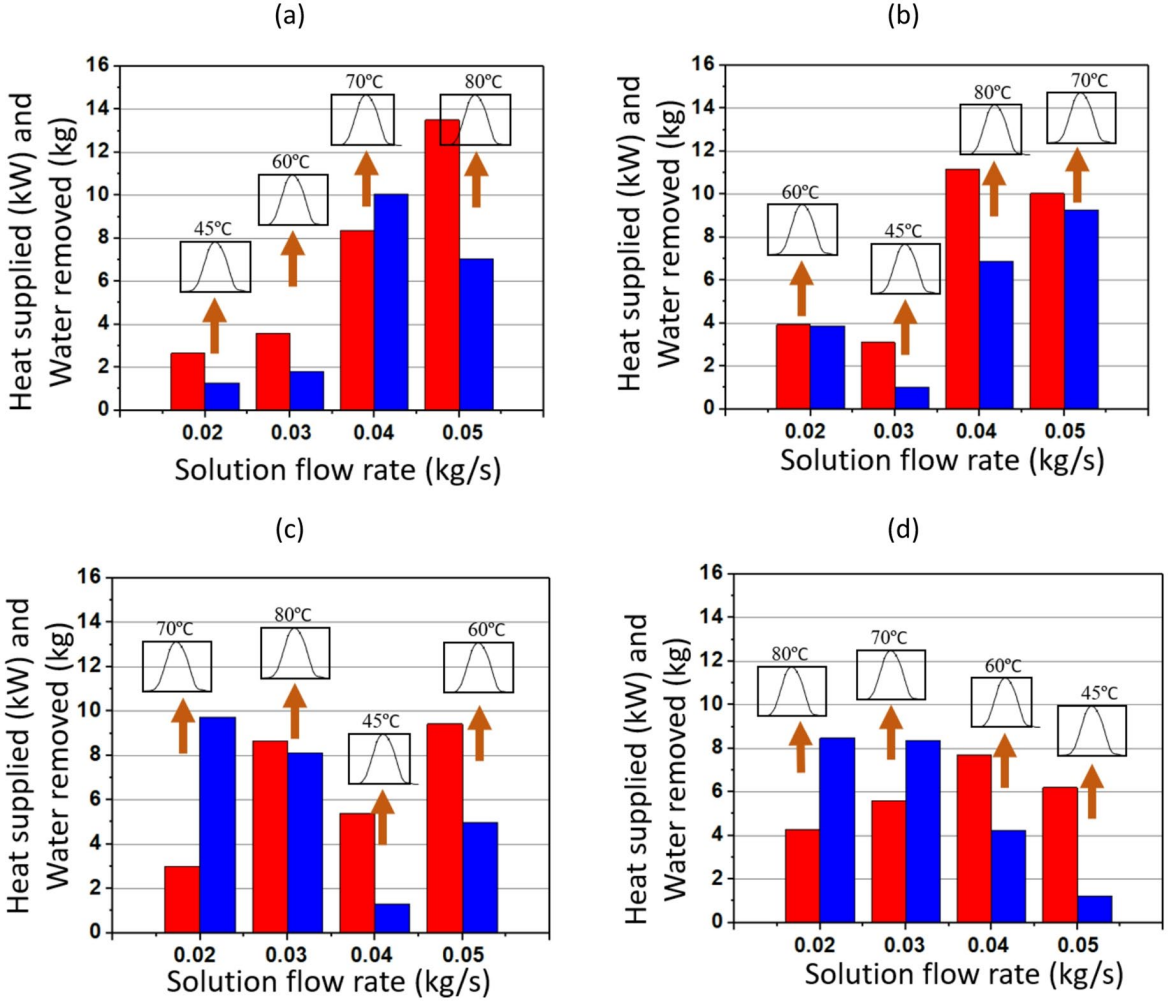
Performance of TCF energy network for ṁ_a_ of (**a**) 0.07 kg/s, (**b**) 0.1 kg/s, (**c**) 0.13 kg/s, and (**d**) 0.14 kg/s (where the heat supplied and water removed are represented by red and blue bars, respectively).

Figs. 11 and 12 illustrate the performance of a TCF energy network using a Gaussian-type waste heating profile for heat recovery under varying ṁ_a_ and ṁ_s_. The process is based on heat absorption by a desiccant solution, whereby water is desorbed depending on the heat supplied. The network performance is governed by the interaction between air and solution flows, which together dictates the rates of heat exchange and moisture removal. As ṁ_a_ decrease, the contact time between air and desiccant solution increases, leading to more efficient heat and mass transfer. The temperature gradient plays a crucial role: higher temperatures (e.g. 80 °C) increase the vapour pressure difference between the air and the solution, enhancing desorption and allowing more water to be removed. Conversely, at lower temperatures (e.g. 45 °C), the driving force for water removal is weaker, resulting in less efficient moisture extraction.

In Fig. 11 (a), when ṁ_s_ is 0.05 kg/s and ṁ_a_ is 0.07 kg/s, the heat supplied at 80 °C reaches a maximum of almost 13.5 kW, with water removal of nearly 7 kg. At 80 °C, the network consistently shows higher heat supplied. As ṁ_a_ increases to 0.10 kg/s, the heat supplied decreases, particularly at 45 °C and 60 °C, where the water removal is also significantly lower than at higher temperatures. As shown in Fig. 11 (c), for ṁ_a_ of 0.14 kg/s and T_H_ of 60 °C, the heat supplied is around 8 kW, while the water removed drops to about 4 kg, reflecting the impact of moderate temperature on performance.

In Fig. 12, the focus shifts to different ṁ_a_ while varying ṁ_s_. Similar to Fig. 11, the trend indicates that higher temperatures generally lead to higher heat supplied and greater water removal at a given ṁ_s_. For instance, in Fig. 12 (a) with ṁ_a_ of 0.07 kg/s and T_H_ of 80 °C, the heat supplied reaches around 14 kW, and water removal peaks at about 7 kg. The temperature variation from 45 °C to 80 °C illustrates the strong temperature dependence of the TCF network’s performance. The highest temperature (80 °C) does not always outperform lower temperatures across all flow rates in both heat supplied and water removed, rather 70 °C outperforms that underscoring the importance of maintaining optimum temperature conditions for maximum waste heat recovery.

**Fig. 13:**
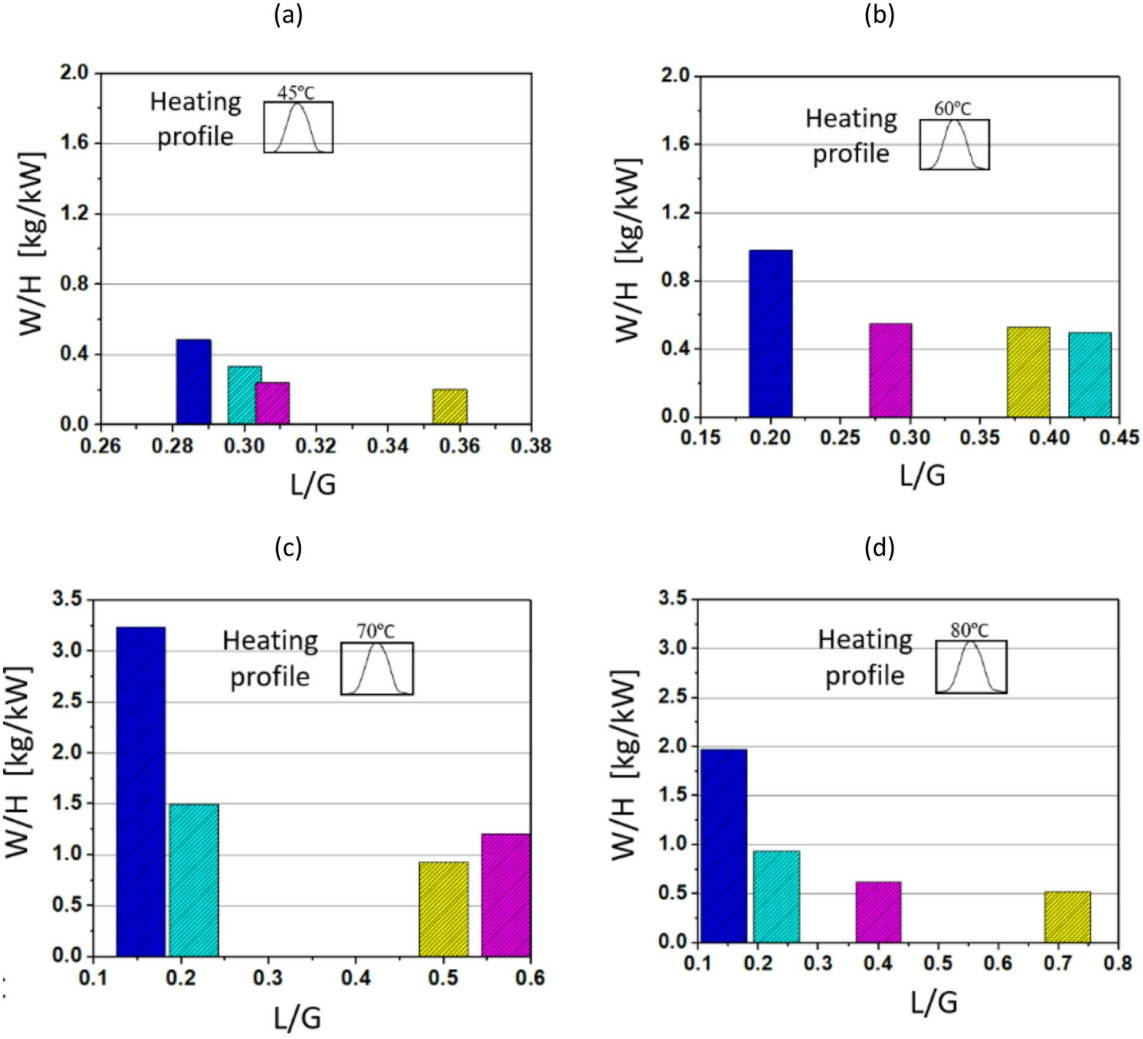
Performance of TCF energy network over L/G ratios with maximum Gaussian T_H_ of (**a**) 45 °C, (**b**) 60 °C, (**c**) 70 °C, and (**d**) 80 °C.

Fig. 13 illustrates the performance of the TCF energy network, showing the relationship between W/H and L/G for varying maximum temperatures of Gaussian heating profile.

In Fig. 13 (a), with a T_H_ of 45 °C, the highest W/H is achieved for a L/G ratio of 0.28, where the value reaches around 0.42 kg/kW. As the L/G ratio increases to 0.3, W/H drops to about 0.32 kg/kW, followed by a further reduction as L/G continues to rise. This indicates that at lower temperatures the network performs more effectively at lower L/G ratios, as excess liquid flow beyond 0.30 may lead to reducing returns in water removal performance due to limited mass transfer from the desiccant. In Fig. 13 (b) where T_H_ is raised to 60 °C, the highest W/H ratio is observed at an L/G ratio of 0.2, reaching a value close to 1 kg/kW. This is significantly higher than the performance at T_H_ of 45 °C, highlighting the positive impact of increased heating temperatures on network efficiency. As the L/G ratio increases, W/H decreases, falling below 0.5 kg/kW at an L/G of 0.38. In Fig. 13 (c), the system shows a significantly higher water removal amount at a T_H_ of 70 °C with W/H peaking at around 3.2 kg/kW for an L/G ratio of 0.15. This demonstrates a pronounced improvement in performance at this higher heating temperature. Similar to Figs. 13 (a) and (b), increasing the L/G ratio leads to a notable reduction in performance, with the W/H ratio falling to about 0.9 kg/kW at L/G ratio of 0.5. In Fig. 13 (d) with T_H_ further increased to 80 °C, the highest water removal is observed for a L/G ratio of 0.12, where W/H reaches about 2 kg/kW. Although this value is lower than the performance at T_H_ of 70 °C, the network still demonstrates relatively high efficiency compared with T_H_ of 45 °C and 60 °C. However, as the L/G ratio increases, W/H declines steeply, dropping to less than 0.6 kg/kW at L/G of 0.68. Overall, the results indicate that as the L/G ratio increases, the W/H ratio decreases, primarily because higher liquid flow requires a larger heat load to regenerate the desiccant solution. Furthermore, it is observed that at higher regeneration temperatures (e.g., T_H_ of 80 °C), the performance declines compared to 70 °C, which could be due to the air becoming saturated at the higher temperature, thereby limiting further moisture desorption.

The observed decline in performance at 80 °C compared with 70 °C in Fig. 13 is primarily associated with increased thermal losses and reduced effective heat utilisation. At higher regeneration temperatures, convective and radiative heat losses to the surroundings become more significant, while the marginal increase in desorption driving force diminishes. Also, elevated temperatures can locally reduce solution residence time and weaken air–solution contact efficiency, limiting further performance gains.

#### Network with RTO heating profile

**Fig. 14:**
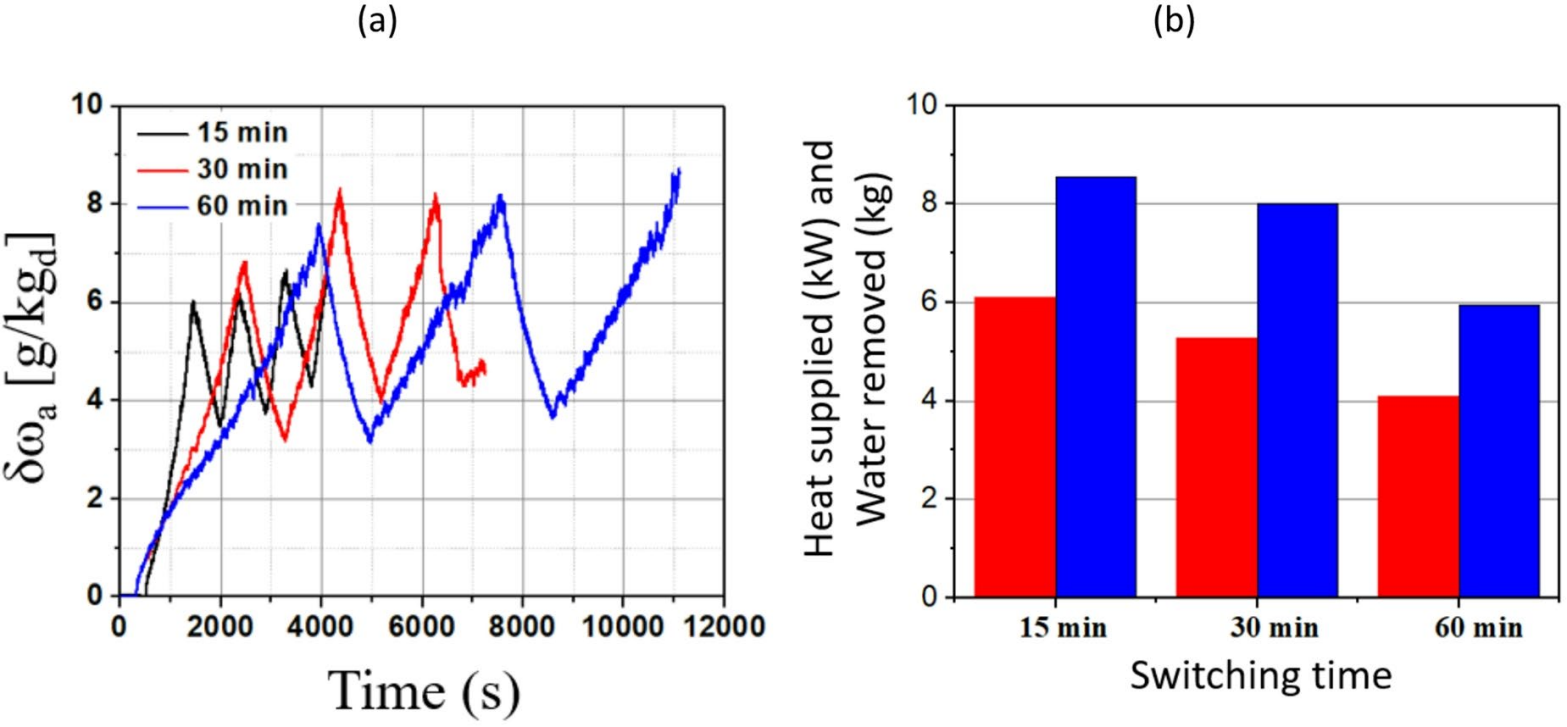
Performance of TCF energy network with RTO waste heating profile and different switching times showing (**a**) δω_a_ over time and (**b**) the comparison of water removal and heat supply (where the heat supplied and water removed are represented by red and blue bars, respectively).

The performance of the TCF energy network using an RTO waste heating profile is illustrated in Fig. 14. In Fig. 14 (a), δω_a_ is plotted over time for three switching times: 15, 30, and 60 min. For the 15-min switching time, a sharp and frequent rise in δω_a_ is observed, with peak values reaching close to 6.6 g/kg_da_, indicating frequent regeneration cycles and rapid changes in humidity levels. In contrast, the 60-min switching time exhibits a slower but more sustained increase in δω_a_, with a maximum of around 8.9 g/kg_da_. The intermediate 30-min switching time shows the highest peak under 5,000 s.

Fig. 14 (b) compares the heat supplied and water removed for each switching time. For the 15-min switching time, the heat supplied is highest (at around 6 kW) and the water removed is also substantial (at about 9 kg). For the 30-min switching time, the heat supplied drops to around 5 kW, whereas the water removal remains high, at about 8 kg, suggesting a more favourable balance between energy input and water removal. For the 60-min switching time, both the heat supplied and the water removed decrease to about 4 kW and 6 kg, respectively. This indicates that longer switching times reduce energy input but also lead to less water removal, highlighting a trade-off between operational efficiency and network performance.

### Comparison of TCF energy networks with different heating profiles

**Fig. 15:**
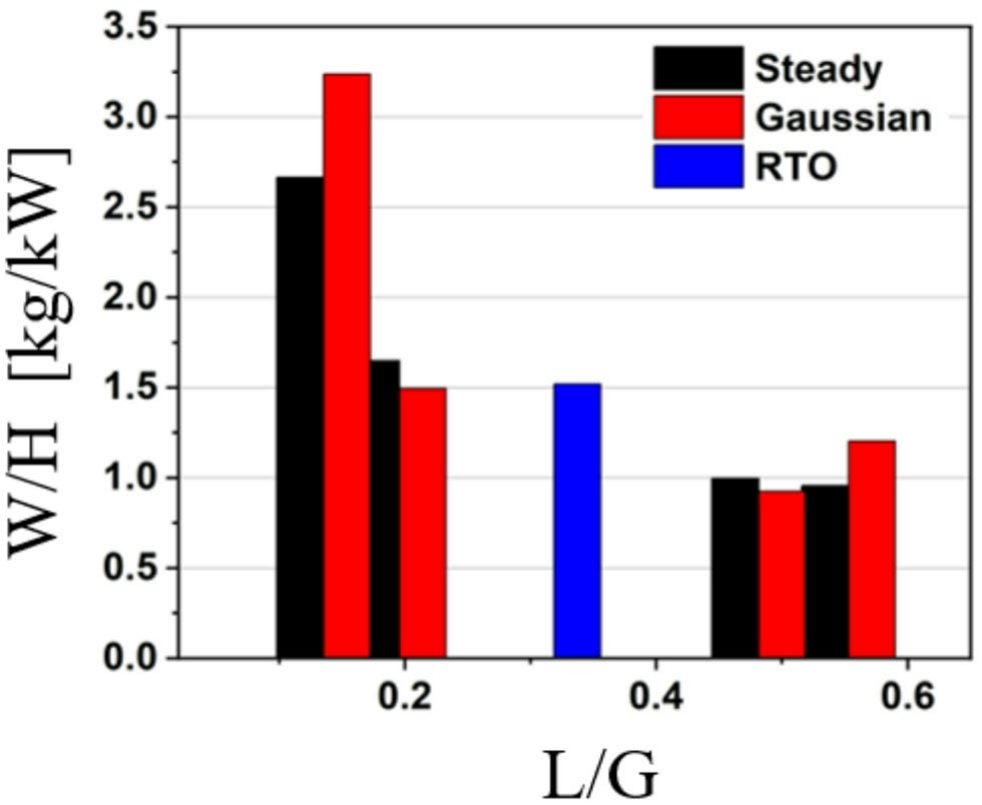
Performance comparison of TCF energy network with heating profiles.

To compare the different heating profiles in terms of achieving higher water removal with minimum heat input, a performance assessment was carried out. Fig. 15 presents the performance of the TCF energy network under three waste heating profiles (i.e. steady, Gaussian, and RTO) at a maximum T_H_ of 70 °C. It shows W/H as a function of L/G for each heating profile, providing insight into how different waste heat sources affect network performance.

For the steady heating profile (black bars), the W/H ratio is highest at an L/G ratio below 0.2, with a value of around 2.7 kg/kW, showing that steady heat input leads to efficient water removal at low L/G. However, as the L/G ratio increases to 0.46 and 0.53, the W/H ratio drops significantly to about 1 kg/kW and 0.9 kg/kW, respectively. The Gaussian heating profile (red bars) exhibits the highest W/H ratio at an L/G of around 0.2, reaching approximately 3.3 kg/kW and slightly outperforming the steady profile. This indicates that Gaussian heating is particularly effective at lower L/G ratios, possibly because the temporal variation in heat input enhances the thermal regeneration of the desiccant. As with the steady profile, W/H decreases with increasing L/G, falling to around 1.2 kg/kW at L/G of 0.56, indicating a consistent decline in performance as the desiccant flow rate increases. In contrast, the RTO heating profile (blue bars) shows a different pattern. At an L/G of 0.32, the W/H ratio is lower (at about 1.5 kg/kW), indicating that the RTO profile is less effective at low L/G ratios than the steady and Gaussian profiles. Overall, the Gaussian profile offers the highest water removal per unit heat input at lower L/G ratios, followed by the steady profile, while the RTO profile performs less favourably in these range. The superior performance of the Gaussian heating profile at low L/G ratios can be attributed to its time-concentrated heat delivery, which better matches the transient mass-transfer demand of the desiccant film. At low liquid flow rates, the Gaussian profile provides a higher instantaneous regeneration potential during the peak period, enhancing desiccant reconcentration without excessive sensible heating of the air stream. In contrast, steady and RTO-type profiles distribute heat more uniformly over time, leading to lower peak driving potential under liquid-limited conditions. This comparison highlights the importance of matching the waste heating profile to the operating conditions to optimise the performance of the TCF energy network.

### Desiccant simulator for dynamic modelling

A simulator is developed by considering the network’s inlet and outlet parameters to predict performance parameters, such as δω_a_ and ε_m_, in terms of the network’s independent parameters. As observed in Sect. 3, the regeneration performance is non-linear. Therefore, the number of hidden layers and neurons in the simulator must be adjusted to train the network and obtain an accurate prediction model. After successful preprocessing, the datasets are prepared for training, and a novel lightweight AI-MLP-based simulator is proposed.

**Fig. 16:**
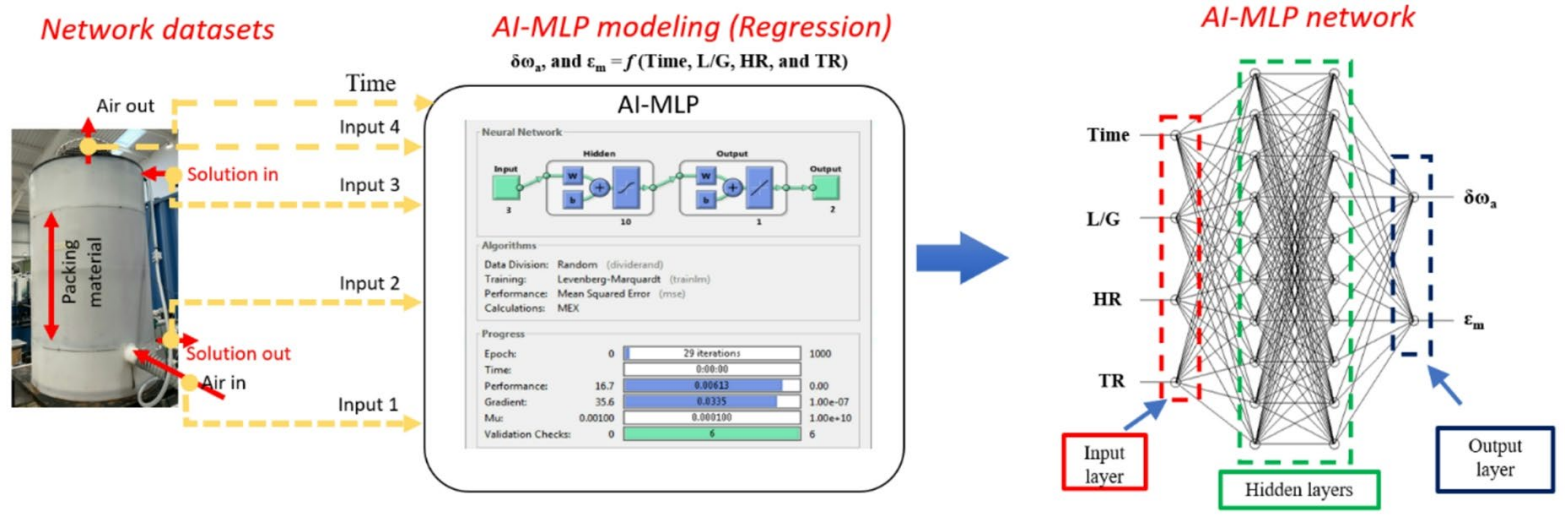
TCF energy network simulator for performance prediction.

A detailed schematic of the simulator used to analyse network performance is shown in Fig. 16. On the left, the regenerator component of the test rig is depicted, consisting of a column packed with structured packing material. Time, air and solution parameters are treated as key inputs, labelled Input 1 to Input 4, and collectively form the regeneration dataset used in the AI-MLP model. The middle part of Fig. 16 shows the AI-MLP architecture, consisting of an input layer, multiple hidden layers (which are varied in number), and an output layer. The input layer includes nodes corresponding to time, L/G, HR, and TR, which are fed into the network. The model is trained to predict two specific outputs, δω_a_ and ε_m_, as functions of time, L/G, HR, and TR. The right part of Fig. 16 presents the final AI-MLP architecture, illustrating the flow of data through the network. The inputs are processed through two hidden layers, where interconnected neurons transform the input data into a set of outputs. The output layer then produces the final predictions (δω_a_ and ε_m_) based on the learned relationships.

**Fig. 17:**
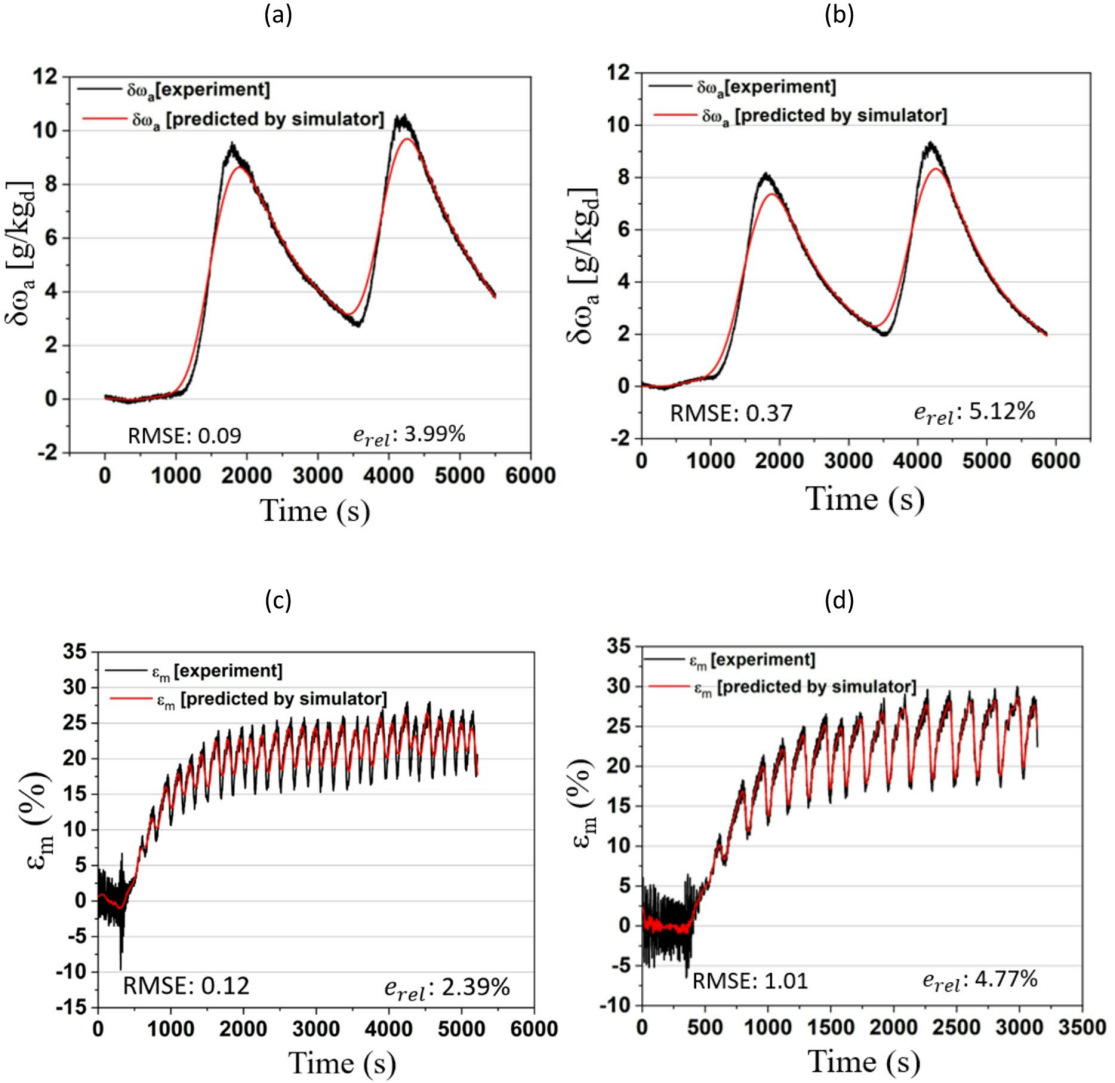
Comparison of experimental datasets and simulator prediction for TCF energy network under different operating conditions: Gaussian with (**a**) L/G of 0.15 and (**b**) 0.21 and steady with (**c**) L/G of 0.31 and (**d**) 0.39.

Fig. 17 illustrates the performance of the simulator designed to predict the TCF-based energy network. Fig. 17 (a) shows the network under a Gaussian heat profile with a L/G ratio of 0.15. The time-averaged predicted and experimental datasets for δω_a_ are closely aligned, with a RMSE of 0.09 and *e*_*rel*_ of 3.99%. This close alignment indicates that the AI-MLP simulator is highly robust under these conditions. Conversely, Fig. 17 (b), corresponding to a Gaussian profile with L/G ratio of 0.21, shows a slightly higher RMSE of 0.37 and a relative error of 5.12%. Although the AI-MLP predictions still follow the experimental data reasonably well, the increased error metrics suggests a modest reduction in predictive accuracy compared with Fig. 17 (a). Fig. 17 (c) presents the network’s performance at a steady profile with L/G ratio of 0.31, showing both the experimental and time-averaged predicted datasets for ε_m_. The AI-MLP model again performs well, with RMSE of 0.12 and *e*_*rel*_ of 2.39%, indicating that the model is reliable under these steady conditions. However, Fig. 17 (d) illustrating the network’s behaviour for a steady heating profile with L/G ratio of 0.39, shows a larger deviation between the time-averaged predicted and experimental data (RMSE of 1.01 and *e*_*rel*_ of 4.77%), indicating a noticeable decline in predictive accuracy as the operating conditions change, particularly at this higher L/G ratio.

## Conclusions

This study investigated the performance of a thermochemical fluid (TCF) based energy network designed for waste heat recovery, utilising three distinct heating profiles, i.e. Gaussian, steady, and regenerative thermal oxidiser (RTO). Based on the experimental results and simulations, the following conclusive points can be drawn:

•Optimal energy recovery is achieved at lower solution mass flow rates, particularly at 0.03 kg/s, where the network reaches its highest effectiveness, recovering about 30% of the potential energy. 

•Increasing the heating temperature significantly enhances the moisture recovery (δωₐ) of the TCF network, with peak values of up to 4.3 g/kgda observed at 80°C. In addition, higher temperatures reduce the system's sensitivity to variations in the liquid-to-gas (L/G) ratio, further improving operational stability across a range of conditions.

•The water removal to heat supplied ratio (W/H) decreases consistently as the L/G ratio increases, indicating reduced performance at higher desiccant flow rates. This trend was observed across all heating profiles, underscoring the need to optimise L/G ratios for improved network efficiency.

•Among the tested profiles, the Gaussian heating profile delivers the highest W/H ratio at lower L/G ratios, peaking at around 3.3 kg/kW at an L/G of 0.2. This suggests that variable heat input, characteristic of the Gaussian profile, enhances the desiccant's moisture removal capacity and should be considered for scenarios requiring optimised water removal efficiency.

•The AI-based multi-layer perceptron (MLP) simulator developed for this study shows strong predictive accuracy under lower L/G ratios and both Gaussian and steady heating profiles, with minimal error (RMSE as low as 0.09). However, as the L/G ratio increases, the model's accuracy diminishes slightly, indicating the need for further refinement in predicting performance under more dynamic or high-flow conditions.

These findings provide critical insights into the performance and optimisation of TCF energy networks, particularly in relation to flow rate management, heating profiles, and the role of advanced predictive models. By fine-tuning these factors, the efficiency of sustainable thermal management systems can be significantly enhanced. A detailed investigation of packing wetting dynamics and their direct impact on system performance will be addressed in future studies.

## Figures and Tables

**Fig. B1:**
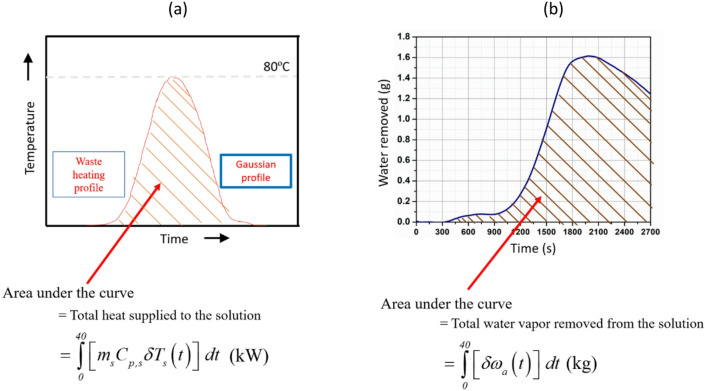
(**a**) Temperature curve over time follows a Gaussian pattern (**b**) water removal curve over time.

## Data Availability

The supporting research dataset is published in the Durham University Research Data Repository. DOI: http://doi.org/10.15128/r2j96020698.

## Appendix A: thermodynamic equations for the working fluids

The following thermodynamic functions for the water, the moist air and the TCF (CaCl_2_) are used^39,41^:

- Vapour pressure of water, *P*_*H2O*_ (kPa):

A.1 $$\begin{aligned}\:{P}_{H2O}=\:{P}_{c}\cdot\:\mathrm{e}\mathrm{x}\mathrm{p} \Big[(\frac{T_c}{T_K})\cdot\:(7.85951783\cdot\:{\uptau\:}+1.84408259\cdot\:{{\uptau\:}}^{1.5}-11.7866497{\cdot\:{\uptau\:}}^{3}\\+22.6807411{\cdot\:{\uptau\:}}^{3.5}-15.9618719{\cdot\:{\uptau\:}}^{4}+1.80122502{\cdot\:{\uptau\:}}^{7.5} )\Big]\end{aligned}$$

where *P*_*C*_ is the critical pressure of water (22,060 kPa), *T*_*K*_ is the temperature of water expressed in Kelvin and *T*_*c*_ is the critical temperature of water in Kelvin (647.096 K), while *τ* is defined as:

A.2 $$\:{\uptau\:}=\:1-\frac{{T}_{K}}{{T}_{C}}$$

- Density of water, *ρ*_*H2O*_ (kg/m^3^):

A.3 $$\begin{aligned}\:{\rho\:}_{H2O}=322\cdot\:(1+1.993771843\cdot\:{{\uptau\:}}^{1/3}+1.0985211604\cdot\:{{\uptau\:}}^{2/3}-0.5094492996\cdot\:{{\uptau\:}}^{5/3}\\-1.761912427\cdot\:{{\uptau\:}}^{16/3}-44.9005480267\cdot\:{{\uptau\:}}^{43/3}-723\:692.2618632\cdot\:{{\uptau\:}}^{110/3})\end{aligned}$$

- Specific heat capacity of water, *c*_*p, H2O*_ (kJ/kg⋅°C):

A.4 $$\:{c}_{p,H2O}=\:88.7891-120.1958\cdot\:\:{\theta\:}^{0.02}-16.9264\cdot\:\:{\theta\:}^{0.04}+52.4654\cdot\:\:{\theta\:}^{0.06}+0.10826\cdot\:\:{\theta\:}^{1.8}+0.46988\cdot\:\:{\theta\:}^{8}$$

where θ is defined as:

A.5 $$\:{\uptheta\:}=\frac{{T}_{K}}{228}-1$$

- Humidity ratio, *ω* (kg_H2O_/kg_dry, air_):

A.6 $$\:\omega\:=\:0.6219907\cdot\:\frac{{P}_{v}}{P-{P}_{v}}$$

where P and *P*_*v*_ is the total and vapour pressure of the air (kPa).

- Enthalpy of moist air, *h*_*air*_ (kJ/kg):

A.7 $$\:{h}_{air}=\:1.006\cdot\:{T}_{air}+\omega\:\cdot\:\left({c}_{p,H2O}\cdot\:{T}_{air}+2501\right)$$

where *T*_*air*_ is the dry bulb temperature of the moist air (°C).

- Vapour pressure of CaCl_2_ solution, *P* (kPa): Antoine equation is used for its estimation, as shown in Eq. (A.8):

A.8 $$\:{log}P=A\left(m\right)+\frac{B\left(m\right)}{T\left(K\right)}+\frac{C\left(m\right)}{{T}^{2}\left(K\right)}$$

where the parameters A, B, and C are the cubic functions of concentration (m) of the electrolyte and are calculated from the relations:

A.9 $$\:A\left(m\right)={A}_{0}+{A}_{1}m+{A}_{2}{m}^{2}+{A}_{3}{m}^{3}$$

A.10 $$\:B\left(m\right)={B}_{0}+{B}_{1}m+{B}_{2}{m}^{2}+{B}_{3}{m}^{3}$$

A.11 $$\:C\left(m\right)={C}_{0}+{C}_{1}m+{C}_{2}{m}^{2}+{C}_{3}{m}^{3}$$

where the parameters A_0_ - A_3_, B_0_ - B_3_, and C_0_ - C_3_ were determined from the experimental vapour pressure data by the least-squares method. The calculated vapour pressures from Eqs (A.8) were in good agreement with the experimental results for the desiccant solution, and the average deviation was within 1.0%^42^. Table A.1 summarises the best-fit parameters in Eqs (A.9–A.11).

**Table 6:** Best-fit parameters in Eqs (A.9-A.11).

	A0	A1	A2	A3	B0	B1
CaCl_2_ + H_2_O	7.133946	0.06666	0.017125	-0.00304	-1647.33	-65.8949

- Density of CaCl_2_ solution, *ρ*_*TCF*_ (kg/m^3^):

$$\:{\rho\:}_{TCF}=\:{\rho\:}_{H2O}\cdot\:\left(1\:+\:0.836014\:.\:{\upepsilon\:}\:\:-0.4363\:.\:{\upepsilon\:}^2+0.105642\: .{\upepsilon\:}^3\right)$$

where *ε* is defined as:

$$\:\epsilon\:=\:\frac{x}{1-x}$$

- Specific heat capacity of CaCl_2_ aqueous solution, *c*_*p, TCF*_ (kJ/kg⋅°C) :

$$\:{c}_{P,TCF}={c}_{p,H2O}\cdot\:\left(1-{f}_{1}-{f}_{2}\right)$$

where *f*_*1*_ is defined as:

$$\:{f}_{1}=1.63799\cdot\:x-1.69002\cdot\:{x}^{2}-1.05124\cdot\:{x}^{3}$$

while the function *f*_*2*_ is defined as:

$$\:{f}_{2}=58.5225\cdot\:{\theta\:}^{0.02}-105.6343\cdot\:{\theta\:}^{0.04}+47.7948\cdot\:{\theta\:}^{0.06}$$

## Appendix B: heat supplied and water removal calculation procedure

The input profile in Fig. B1 (a) illustrates the temperature curve over time, which follows a Gaussian pattern.

The area covered in Fig. B1 (a) represents the total heat supplied to the desiccant solution, whereas Fig. B1 (b) represents the total water removal from the desiccant solution due to the respective heat supplied. This is area is estimated using Origin Pro software.

## Nomenclature

A: Amplitude of heating profile(°C)
c_p_: Specific heat capacity(kJ/(kg K))
h: Convective heat transfer coefficient(W/(m²K))
H: Heat supplied to the regenerator(kJ)
ṁ_a_: Air mass flow rate(kg/s)
ṁ_s_: Solution mass flow rate(kg/s)
Q: Heat transfer rate(W)
t: Time(s)
t_c_: Centre time of Gaussian profile(s)
T: Temperature(°C)
T_base_: Baseline inlet air temperature(°C)
W: Water removed from air(kg)
W/H: Water removal to heat supplied ratio(kg/kJ)

## Greek Symbols

β: Packing height(m)
δω_a_: Air humidity ratio difference(g/kg_da_)
δ: Flute height (m)
ε_m_: Moisture effectiveness(–)
σ: Standard deviation of Gaussian profile(s)
τ: Period of heating cycle(s)
ω_a_: Air humidity ratio(g/kg_da_)
$$\phi$$: Packing diameter(m)
ω_s_: Solution humidity ratio in equilibrium with air(g/kg_da_)
ω_a,in_: Inlet air humidity ratio(g/kg_da_)
ω_a,out_: Outlet air humidity ratio(g/kg_da_)

## Abbreviations

AH: Air heater
AI: Artificial intelligence
ANN: Artificial neural network
CaCl_2_: Calcium chloride
CO_2_: Carbon dioxide
D: Dehumidifier
DOE: Design of experiment
F: Fan
GUI: Graphical user interface
H: Heater
HS: Heat sink
LD: Liquid desiccant
LiCl: Lithium chloride
L/G: Liquid-to-gas mass flow ratio
MLP: Multi-layer perceptron
P: Pump
PCM: Phase change material
R: Regenerator
RMSE: Root mean squared error
RTO: Regenerative thermal oxidiser
T: Tank
TCF: Thermochemical fluid
